# A New Mechanism for Ribosome Rescue Can Recruit RF1 or RF2 to Nonstop Ribosomes

**DOI:** 10.1128/mBio.02436-18

**Published:** 2018-12-18

**Authors:** Tyler D. P. Goralski, Girish S. Kirimanjeswara, Kenneth C. Keiler

**Affiliations:** a Department of Biochemistry and Molecular Biology, The Pennsylvania State University, University Park, Pennsylvania, USA; b Department of Veterinary and Biomedical Science, The Pennsylvania State University, University Park, Pennsylvania, USA; University of Rochester; University of Virginia Health System; Stonybrook University

**Keywords:** *Francisella tularensis*, ribosomes, Tn-seq, *trans*-translation

## Abstract

Bacterial ribosomes frequently translate to the 3′ end of an mRNA without terminating at an in-frame stop codon. In all bacteria studied to date, these “nonstop” ribosomes are rescued using *trans*-translation. Genes required for *trans*-translation are essential in some species, but other species can survive without *trans*-translation because they express an alternative ribosome rescue factor, ArfA or ArfB. Francisella tularensis cells lacking *trans*-translation are viable, but F. tularensis does not encode ArfA or ArfB. Transposon mutagenesis followed by deep sequencing (Tn-seq) identified a new alternative ribosome rescue factor, now named ArfT. *arfT* can be deleted in wild-type (wt) cells but not in cells that lack *trans*-translation activity. Overexpression of ArfT suppresses the slow-growth phenotype in cells lacking *trans*-translation and counteracts growth arrest caused by *trans*-translation inhibitors, indicating that ArfT rescues nonstop ribosomes *in vivo*. Ribosome rescue assays *in vitro* show that ArfT promotes hydrolysis of peptidyl-tRNA on nonstop ribosomes in conjunction with F. tularensis release factors. Unlike ArfA, which requires RF2 for activity, ArfT can function with either RF1 or RF2. Overall, these results indicate that ArfT is a new alternative ribosome rescue factor with a distinct mechanism from ArfA and ArfB.

## Abstract (precis)

Francisella tularensis is a highly infectious intracellular pathogen that kills more than half of infected humans if left untreated. F. tularensis has also been classified as a potential bioterrorism agent with a great risk for deliberate misuse. Recently, compounds that inhibit ribosome rescue have been shown to have antibiotic activity against F. tularensis and other important pathogens. Like all bacteria that have been studied, F. tularensis uses *trans*-translation as the main pathway to rescue stalled ribosomes. However, unlike most bacteria, F. tularensis can survive without any of the known factors for ribosome rescue. Our work identified a F. tularensis protein, ArfT, that rescues stalled ribosomes in the absence of *trans*-translation using a new mechanism. These results indicate that ribosome rescue activity is essential in F. tularensis and suggest that ribosome rescue activity might be essential in all bacteria.

## INTRODUCTION

Bacterial ribosomes frequently translate to the 3′ end of an mRNA that does not have a stop codon (1–3). These “nonstop” ribosomes cannot terminate translation using one of the canonical termination factors, RF1 or RF2, because they require interactions with the stop codon to activate peptidyl-tRNA hydrolysis (4, 5). Data from Escherichia coli indicate that 5% to 10% of ribosomes that initiate translation do not terminate translation at a stop codon on the mRNA and instead have to be rescued (2, 3). The primary ribosome rescue pathway in all bacteria that have been investigated is *trans*-translation (1, 2, 6). In this pathway, the transfer-messenger RNA (tmRNA)–SmpB complex recognizes a nonstop ribosome and uses a tRNA-like domain of tmRNA and a specialized reading frame within tmRNA to tag the nascent polypeptide for degradation and release the nonstop ribosome (1, 2, 6, 7). Genes encoding tmRNA (*ssrA*) and SmpB (*smpB*) have been identified in >99% of sequenced bacterial genomes, and in some species these genes are essential (1, 8). In other species, *trans*-translation is not essential due to the presence of an alternative ribosome rescue factor, ArfA or ArfB (9, 10). ArfA is a short protein that inserts its C-terminal tail into the mRNA channel of nonstop ribosomes and rescues them by activating RF2 to hydrolyze the peptidyl-tRNA (10–16). ArfA does not interact with the RF2 residues that recognize a stop codon but instead binds a different part of RF2 to stabilize the active conformation and promote peptidyl-tRNA hydrolysis (13–16). These interactions cannot be made with RF1, so ArfA functions only in conjunction with RF2 (11-16). ArfB also binds the empty mRNA channel of nonstop ribosomes with its C-terminal tail, but ArfB contains an RF1-like catalytic domain that can hydrolyze peptidyl-tRNA on nonstop ribosomes in the absence of RF1 or RF2 (17–20). In bacteria that have a functional ArfA or ArfB, deletions of *ssrA* and the gene encoding the alternative ribosome rescue factor are synthetically lethal, indicating that these species require at least one mechanism for rescuing nonstop ribosomes (9, 10).

Although *ssrA* has been deleted from the pathogen F. tularensis (21), no homologues of *arfA* or *arfB* have been found in sequenced F. tularensis genomes. F. tularensis has a reduced genome size and a life cycle that is different from that of many other bacteria, so it is possible that ribosome rescue is not essential. Alternatively, F. tularensis may have an alternative ribosome rescue system that is sufficiently different from ArfA and ArfB that it cannot be identified by homology searches. F. tularensis is a Gram-negative, facultative intracellular bacterium responsible for the vector-borne zoonosis tularemia (22–28). Pneumonic tularemia is infectious at ≤10 CFU (of aerosolized bacteria) and has a 60% mortality rate if left untreated (22–27). F. tularensis has been classified as a tier 1 select agent by the CDC because the bacteria can be easily propagated and disseminated as an aerosol, making the threat of a bioterrorist attack with an antibiotic-resistant strain of F. tularensis a significant concern (26, 27).

To determine if ribosome rescue is essential in F. tularensis, we screened for an alternative ribosome rescue factor using transposon mutagenesis followed by deep sequencing (Tn-seq) in the F. tularensis subsp. *holarctica* live vaccine strain (LVS). One gene, F. tularensis 0865 (*FTA_0865*), renamed here as alternative ribosome rescue factor T (ArfT), was found to be essential in cells lacking *trans*-translation but not in wild-type F. tularensis. We show that ArfT can rescue nonstop ribosomes *in vivo* and *in vitro* and that ArfT can function in conjunction with either RF1 or RF2. These data indicate that ribosome rescue is essential in F. tularensis and that ArfT is the first representative of a new family of alternative ribosome rescue factors that can recruit either RF1 or RF2 to nonstop ribosomes.

## RESULTS

### Identification of an alternative rescue factor in F. tularensis.

A published report demonstrated that an F. tularensis strain in which *ssrA* was disrupted by insertion of an LtrB intron (*ssrA*::*LtrB-bp147*) was viable (21). We used reverse transcription-PCR (RT-PCR) to confirm that there was no detectable tmRNA in *ssrA*::*LtrB-bp147* cells (see Fig. S1 in the supplemental material), suggesting either that ribosome rescue is not essential in F. tularensis or that F. tularensis has another mechanism to rescue nonstop ribosomes. Homology searches of the F. tularensis genome using sequences or motifs from ArfA and ArfB did not identify any candidate alternative ribosome rescue factors. Therefore, we took a genetic approach to identify genes that might be involved in an alternative ribosome rescue pathway. If F. tularensis has an unknown alternative ribosome rescue pathway, genes required for the alternative pathway should be essential in *ssrA*::*LtrB-bp147* cells but not in wild-type cells. We used Tn-seq to identify genes that could be disrupted in each strain. Cells from each strain were mutagenized with a Himar1-based transposon (29, 30) and the transposon insertion sites were sequenced. The ratio of the normalized number of insertions in *ssrA*::*LtrB-*bp147 to the normalized number of insertions in wild-type was used to identify genes that were much less fit in strain *ssrA*::*LtrB-bp147* (see Table S1 in the supplemental material).

10.1128/mBio.02436-18.2 FIG S1 No tmRNA is present in *ssrA*::*LtrB-bp147* cells. Quantitative PCR (qPCR) results of amplification of tmRNA from cDNA prepared from wild-type F. tularensis (wt) or the *ssrA*::*LtrB-bp147* strain are shown. Relative florescence unit (RFU) data are plotted as a function of PCR cycle, and the positive amplification threshold is indicated by the black line. These results show that the amount of tmRNA in strain *ssrA*::*LtrB-bp147* was decreased by a factor of >10^8^ compared to the amount in the wild-type strain. Download FIG S1, TIF file, 69.4 MB. Copyright © 2018 Goralski et al. 2018 Goralski et al. This content is distributed under the terms of the Creative Commons Attribution 4.0 International license.

10.1128/mBio.02436-18.5 TABLE S1 Quantification of transposon insertions. Download TABLE S1, PDF file, 0.2 MB. Copyright © 2018 Goralski et al. 2018 Goralski et al. This content is distributed under the terms of the Creative Commons Attribution 4.0 International license.

**FIG 1:**
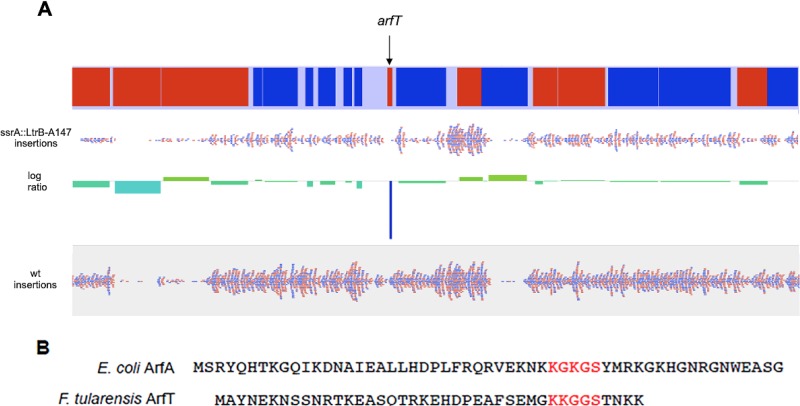
Tn-seq identified ArfT as a candidate alternative ribosome rescue system. (A) Representation of Tn-seq data. The portion of the F. tularensis subsp. *holarctica FTNF002-00* chromosome containing *arfT*, with genes transcribed to the right in red and those transcribed to the left in blue (top), is shown with mapped transposon insertion sites (red and blue dots) in strain *ssrA*::*LtrB-bp147* and wild-type F. tularensis (wt). The number of insertions per gene was normalized to the total number of reads, and the log ratio of the normalized number of insertions was plotted (center) to identify genes that can be deleted in the wild-type strain but not in strain *ssrA*::*LtrB-bp147*. (B) Alignment of E. coli ArfA and ArfT protein sequences. The KGKGS motif that is conserved in ArfA genes and that binds the empty mRNA channel of the ribosome is shown in red, as are the corresponding residues in ArfT.

Among the genes with no insertions in strain *ssrA*::*LtrB-bp147* and typical insertion density in the wild-type strain, *arfT* was a candidate alternative ribosome rescue factor because it shared some characteristics with ArfA and had no annotated function (Fig. 1A). *arfT* encodes a protein of 40 amino acids, whereas mature ArfA has 52 to 55 amino acids, and ArfT contains a stretch of residues near the C terminus that are similar to a conserved KGKGS sequence found in ArfA (Fig. 1B). Structural studies of ArfA indicate the KGKGS sequence binds in the empty mRNA channel of nonstop ribosomes. A tblastn search (31) showed that ArfT homologues are found in other F. tularensis subspecies and in the closely related species F. hispaniensis but not in more distantly related species (see Table S2 in the supplemental material). *arfT* was not previously annotated as an open reading frame in F. tularensis LVS, the Schu S4 strain, or a number of other sequenced F. tularensis strains, but was annotated in F. tularensis subsp. *holarctica* FTNF002-00. For this reason, transposon insertions were mapped to this genome.

10.1128/mBio.02436-18.5 TABLE S2 Bacterial strains with homologues to ArfT. Download TABLE S2, PDF file, 0.1 MB. Copyright © 2018 Goralski et al. 2018 Goralski et al. This content is distributed under the terms of the Creative Commons Attribution 4.0 International license.

### Deletion of *arfT* is synthetically lethal with disruption of *ssrA*.

The Tn-seq data suggested that the absence of both *trans*-translation and ArfT is lethal to F. tularensis cells. This prediction was tested by attempting to produce markerless, in-frame deletions of *arfT* using a two-step recombination procedure (32) in the wild type, *ssrA*::*LtrB-bp147*, and *ssrA*::*LtrB-bp147* with a plasmid-borne copy of *ssrA* expressed from a strong, constitutive promoter (*ssrA*::*LtrB-bp147 pFtssrA*). In the first step of this procedure, a suicide plasmid containing a copy of the *arfT* locus with the *arfT* coding sequence deleted was recombined into the chromosome. The second recombination step eliminates one copy of the *arfT* locus, so cells can retain either the *arfT* deletion or the wild-type *arfT* gene (Fig. S2) (32). The first recombination step was successful in all strains. For the wild-type strain, 20% of the second-step recombinants had the *arfT* deletion, demonstrating that *arfT* is not essential. Deletion of *arfT* did not cause a large defect in growth or morphology (see Fig. 3). For the *ssrA*::*LtrB-bp147* strain, 100 second-step recombinants were screened and all had retained the wild-type copy of *arfT*, indicating that disruption of both *ssrA* and *arfT* was lethal. When a plasmid-borne copy of *ssrA* was present in *ssrA*::*LtrB-bp147* cells, 20% of the second-step recombinants had *arfT* deleted, demonstrating that the synthetic lethal phenotype can be complemented by an ectopic copy of *ssrA.* FTA_0993, a gene that had transposon insertions in both the wild-type and *ssrA*::*LtrB-bp147* strains in the Tn-seq experiment, was successfully deleted from the *ssrA*::*LtrB-bp147* strain (Fig. S2), confirming that *ssrA*::*LtrB-bp147* cells are competent for recombination in the two-step procedure. Taken together, these data demonstrate that deletion of *arfT* is lethal to F. tularensis cells lacking *trans*-translation and indicate that ribosome rescue is required in F. tularensis.

10.1128/mBio.02436-18.3 FIG S2 Deletion of *arfT* is synthetically lethal with deletion of *ssrA*. Diagnostic PCR was performed to determine if secondary recombinants from allelic exchange represented deletions or reversions to wild type. One example of each strain is shown. Lanes 2 to 5 represent PCR products from reactions performed using ArfT_KO primers, and lanes 6 to 9 represent PCR products from reactions performed using 0993_KO primers. The ArfT_KO primers amplified a 690-bp product for the wild type and a 570-bp product for a deletion of *arfT*. The 0993_KO primers amplified a 670-bp product for the wild type and a 330-bp product for deletion mutants. Lanes: 1, DNA marker; 2, wt control DNA; 3, *arfT* deletion in wt; 4, *arfT* deletion in strain *ssrA*::*LtrB-bp147 *+* *pFtssrA; 5, reversion to wild type in strain *ssrA*::*LtrB-bp147*; 6, wt control DNA; 7, FTA_0993 deletion in wt; 8, FTA_0993 deletion in strain *ssrA*::*LtrB-bp147 *+* *pFtssrA; 9, FTA_0993 deletion in strain *ssrA*::*LtrB-bp147*. Download FIG S2, TIF file, 90.2 MB. Copyright © 2018 Goralski et al. 2018 Goralski et al. This content is distributed under the terms of the Creative Commons Attribution 4.0 International license.

### ArfT can recruit either RF1 or RF2 to hydrolyze peptidyl-tRNA on nonstop ribosomes *in vitro*.

**FIG 2:**
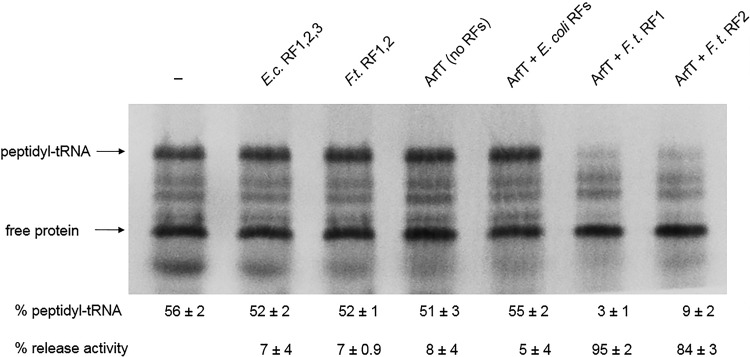
ArfT promotes peptidyl-tRNA hydrolysis on nonstop ribosomes in conjunction with either RF1 or RF2. Gel image of *in vitro* ribosome rescue assays. In vitro transcription/translation assays were programmed with a nonstop DNA template and synthesized protein was labeled by incorporation of ^35^S-methionine. ArfT and release factors were added to individual reaction mixtures in the combinations indicated. Bands corresponding to peptidyl-tRNA and free protein were quantified. The percentage of protein in the peptidyl-tRNA band and the percentage of peptidyl-tRNA that was hydrolyzed compared to a reaction with no release factors or ArfT added (release activity) are shown (± standard deviation). The data represent averages of results from 3 biological replicates. *E.c*., E. coli; *F.t*., F. tularensis.

*In vitro* ribosome rescue assays were performed to assess whether ArfT was capable of rescuing nonstop ribosomes. Nonstop ribosomes were generated by programming a transcription-translation reaction with a gene that does not have a stop codon (dihydrofolate reductase [DHFR]-NS) (Fig. 2) (9). In the absence of ribosome rescue, peptidyl-tRNA was stable on the ribosome and could be observed on protein gels. As expected for nonstop ribosomes, addition of RF1, RF2, and RF3 from E. coli or RF1 and RF2 from F. tularensis did not dramatically decrease the amount of peptidyl-tRNA. Addition of ArfT alone did not promote hydrolysis of the peptidyl-tRNA, indicating that ArfT does not have intrinsic hydrolytic activity to rescue nonstop ribosomes. Likewise, addition of ArfT in conjunction with RF1, RF2, and RF3 from E. coli did not promote peptidyl-tRNA hydrolysis. However, addition of ArfT with F. tularensis RF1 resulted in 95% peptidyl-tRNA hydrolysis and addition of ArfT with F. tularensis RF2 resulted in 84% peptidyl-tRNA hydrolysis (Fig. 2). These data suggest that ArfT can rescue ribosomes by recruiting either RF1 or RF2 to nonstop ribosomes.

### Overexpression of *arfT* rescues the growth defect in cells lacking *trans*-translation.

**FIG 3:**
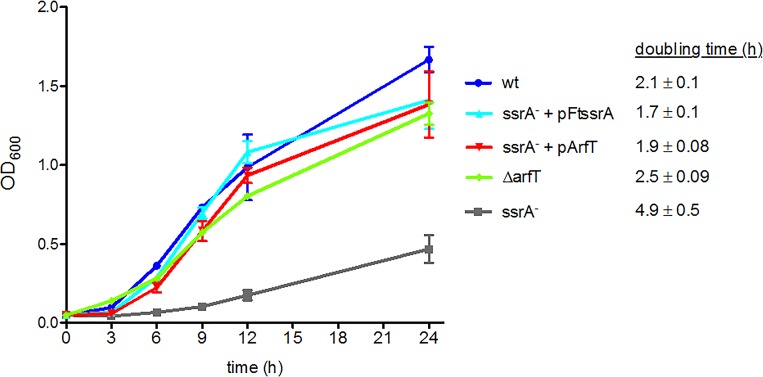
Overexpression of ArfT rescues the growth defect in strain *ssrA*::*LtrB-bp147.* Growth curves of wild-type F. tularensis (wt), the Δ*arfT* strain, and the *ssrA*::*LtrB-bp147* strain (*ssrA*^–^) with and without plasmids expressing *ssrA* (pFtssrA) or *arfT* (pArfT) are shown. Error bars indicate standard deviations. The doubling time for each strain (± standard deviation) is indicated. The data represent averages of results from 3 biological replicates.

**FIG 4:**
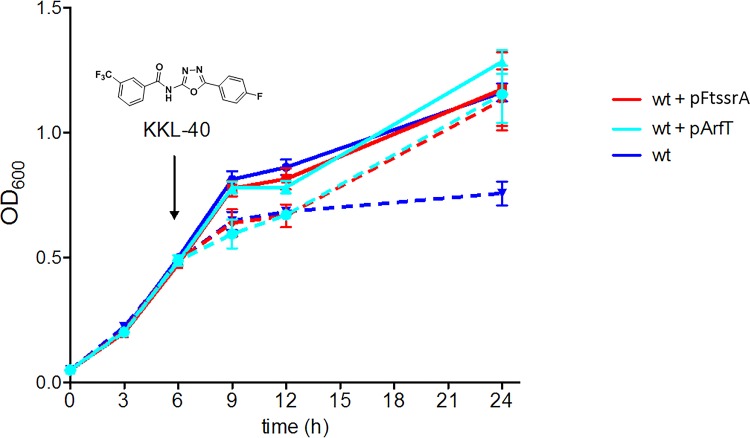
Overexpression of ArfT prevents growth inhibition caused by ribosome rescue inhibitors. Growth curves of wild-type F. tularensis (wt) with and without plasmids expressing *ssrA* (pFtssrA) or *arfT* (pArfT). A ribosome rescue inhibitor, KKL-40 (structure shown), was added to half the cultures after 6 h (indicated by arrow) at 10× MIC. Cultures with KKL-40 are indicated with dotted lines, and cultures with no drug are indicated with solid lines. The data represent averages of results from 3 biological replicates, with error bars indicating the standard deviations.

It was previously reported that the *ssrA*::*LtrB-bp147* strain grows much more slowly than the wild type in liquid culture and that this growth defect could be complemented by expression of *ssrA* from a plasmid (21). To determine whether overexpression of *arfT* could restore normal growth to cells in the absence of *trans*-translation, we cloned *arfT* under the control of the strong, constitutive bacterioferratin (Bfr) promoter on a multicopy plasmid (pArfT) and tested its impact on growth rates. As expected, the *ssrA*::*LtrB-bp147* strain grew substantially more slowly than the wild-type strain, but the *ssrA*::*LtrB-bp147 pFtssrA* strain grew at the same rate as the wild-type strain (Fig. 3). *ssrA*::*LtrB-bp147* cells with pArfT also grew at the same rate as wild-type cells, indicating that multicopy *arfT* genes can suppress the *ssrA* phenotype. pArfT did not increase the growth rate of wild-type cells (Fig. 4). These results suggest that ArfT can rescue nonstop ribosomes in the absence of *trans*-translation.

### Overexpression of ArfT prevents growth arrest due to ribosome rescue inhibitors.

It has been shown that the members of a class of oxadiazole compounds such as KKL-40 inhibit ribosome rescue and arrest the growth of many bacterial species, including F. tularensis (33–35). Overexpression of E. coli ArfA prevents growth arrest by these oxadiazoles in Shigella flexneri, confirming that growth arrest is due to inhibition of ribosome rescue (33, 34). If ArfT had ribosome rescue activity similar to that seen with ArfA, overexpression of ArfT should inhibit growth arrest in F. tularensis mediated by KKL-40. To test this prediction, KKL-40 was added to growing cultures of F. tularensis strains and growth was monitored over 18 h (Fig. 4). As previously observed, addition of KKL-40 resulted in growth arrest of wild-type F. tularensis and the bacteria were unable to recover to normal levels. Addition of KKL-40 to F. tularensis carrying pFtssrA or pArfT caused an initial decrease in the growth rate, but after 18 h the cultures had reached the same density as the wild-type cultures. Because growth inhibition is suppressed by extra ribosome rescue activity in the form of either tmRNA-SmpB or ArfT activity, it is likely that KKL-40 inhibits growth through ribosome rescue and not through off-target effects. These results are consistent with a model in which KKL-40 arrests growth in F. tularensis by binding to nonstop ribosomes and tmRNA-SmpB or ArfT can counteract the effects of KKL-40 by rescuing the ribosomes before KKL-40 binds.

## DISCUSSION

The data described here answer two recently posed outstanding questions regarding ribosome rescue: are there other alternative rescue factor systems, and will ArfA-like systems emerge in bacteria where RF1 is recruited to nonstop ribosomes (36)? The answer to both questions is yes. The data presented here indicate that ArfT has all the characteristics of an alternative ribosome rescue factor in F. tularensis. ArfT has ribosome rescue activity *in vitro* because it can release nonstop ribosomes in conjunction with RF1 or RF2. *In vivo*, deletion of *arfT* is synthetically lethal with disruption of *ssrA*, consistent with ArfT providing essential ribosome rescue activity in the absence of *trans*-translation. Overexpression of ArfT suppresses the slow-growth phenotype in cells lacking *trans*-translation and counteracts growth arrest mediated by a ribosome rescue inhibitor in F. tularensis, indicating that ArfT can perform the same physiological role as *trans*-translation in F. tularensis. These results demonstrate that the presence of ArfT in F. tularensis makes *trans*-translation dispensable and that ribosome rescue activity is essential in F. tularensis.

ArfT has some similarities to ArfA, and the two factors may recognize nonstop ribosomes in the same manner. The C-terminal tail of ArfA binds in the empty mRNA channel of nonstop ribosomes through a number of lysine and arginine residues, including a conserved KGKGS motif (13–16). None of these residues are essential for ArfA activity (16, 37), but replacement of individual residues decreases ribosome rescue activity *in vitro* (16). The KKGGSTNKK sequence near the C terminus of ArfT has an arrangement of positively charged residues that is similar to that in ArfA, suggesting that ArfT may use this sequence to bind the ribosome. SmpB and ArfB also bind in the empty mRNA channel of nonstop ribosomes using positively charged C-terminal tails, but ArfA, SmpB, and ArfB each make different interactions with the mRNA channel (7, 13–20, 37). Because of this variation in binding, structural studies will be required to define the interactions between ArfT and the ribosome.

Despite the similarities in protein size and C-terminal tail sequence between ArfT and ArfA, the observation that ArfT can activate RF1 or RF2 suggests that ArfT may not interact with release factors (RFs) in the same way as ArfA. Cryo-electron microscopy (cryo-EM) analyses of a nonstop ribosome bound to E. coli ArfA-RF2 showed that residues 15 to 31 of ArfA interact with RF2 to stabilize the active conformation of RF2 and promote hydrolysis of the peptidyl-tRNA (13–16). In a key feature of this interaction, ArfA forms a ß-strand that extends the ß-sheet formed by ß4-ß5 of RF2, with F25 of ArfA binding in a hydrophobic pocket formed by V198 and F217 of RF2. Residues in RF2 ß4-ß5 and the SerProPhe (SPF) loop are highly conserved between E. coli RF2 and F. tularensis RF2 (see Fig. S3 in the supplemental material), raising the possibility that ArfT could bind in a manner similar to that seen with ArfA. However, ArfT does not have a hydrophobic residue at the position corresponding to F25 (Fig. 1B). The absence of the V198-F217 pocket in E. coli RF1 has been suggested to be the reason that ArfA does not activate E. coli RF1 (13–16). This region of E. coli RF1 is highly conserved in F. tularensis RF1, and yet ArfT activates F. tularensis RF1 but not E. coli RF1. Therefore, if the interaction between ArfT and RF2 were similar to the interaction between ArfA and RF1, ArfT would have to activate RF1 through a distinct mechanism. Alternatively, ArfT may activate F. tularensis RF1 and RF2 in the same manner as but through a different mechanism than that used by ArfA. Little was known about the interactions among ArfA, RF2, and the ribosome before structural data of the complex were obtained, and similar studies will be required to understand how ArfT can activate both RF1 and RF2.

10.1128/mBio.02436-18.4 FIG S3 Residues in the ArfA-interacting region of E. coli RF2 are conserved in F. tularensis RF2. Alignments of RF2 from E. coli and F. tularensis were performed using blastp (26). Residues in RF2 ß4 (red), ß5 (blue), and the SPF loop (gold) are indicated. Download FIG S3, TIF file, 18.7 MB. Copyright © 2018 Goralski et al. 2018 Goralski et al. This content is distributed under the terms of the Creative Commons Attribution 4.0 International license.

Another likely difference between ArfT and ArfA concerns regulation. The *arfA* gene includes a transcriptional terminator and RNase III cleavage site before the stop codon, such that ArfA protein is made from nonstop mRNA (38, 39). When *trans*-translation is active, the nascent ArfA peptide is tagged and degraded, but when *trans*-translation activity is not available, active ArfA is produced and accumulates in the cell. This genetic arrangement makes ArfA a true backup ribosome rescue system, functioning only when *trans*-translation activity is low or absent (38, 39). The *arfT* gene does not include a transcriptional terminator or an RNase III cleavage site before the stop codon. RT-PCR using a primer corresponding to the final 33 nucleotides of the *arfT* reading frame (including the stop codon) showed that *arfT* mRNA accumulated in wild-type F. tularensis and the *ssrA*-disrupted strain at similar levels (Fig. S4). Although these results do not exclude the possibility that *arfT* mRNA is truncated in the last few codons, the gene product does not appear to be controlled by transcriptional termination and RNase III cleavage in the same manner as ArfA.

10.1128/mBio.02436-18.5 FIG S4 *arfT* mRNA accumulates in F. tularensis. cDNA from the wild type (wt), strain *ssrA*::*LtrB-bp147*, the wild type expressing *arfT* from a plasmid (wt + pArfT), or the wild type expressing *ssrA* from a different plasmid (wt + pFtssrA) was used as the template for qPCR amplification of *arfT*. Relative florescence unit (RFU) data are plotted as a function of the PCR cycle, and the positive amplification threshold is indicated by the black line. Download FIG S4, TIF file, 67.9 MB. Copyright © 2018 Goralski et al. 2018 Goralski et al. This content is distributed under the terms of the Creative Commons Attribution 4.0 International license.

The observations indicating that ArfT interacts with RF1 and is not regulated like ArfA and the overall low sequence similarity between ArfT and ArfA suggest that ArfT evolved independently from ArfA and represents a third different alternative ribosome rescue factor. Our sequence homology searches identified ArfT only in the closely related F. tularensis and F. hispaniensis strains, but the small size of ArfT makes more distant homologues difficult to identify with this method. Characterization of the ArfT residues required for interaction with RF1 and RF2 will allow more-specific searches for ArfT in other species. The number of different ribosome rescue mechanisms discovered to date suggests that the problem presented by nonstop ribosomes has been solved many times throughout evolution, and more alternative ribosome rescue factors may yet be discovered. It is not yet clear what conditions would limit *trans*-translation activity enough that an alternative ribosome rescue factor would be needed. However, such conditions must exist in a wide variety of environments. Alternative ribosome rescue factors have been selected for in enteric bacteria such as E. coli, which has ArfA; aquatic bacteria such as C. crescentus, which has ArfB; and intracellular pathogens such as F. tularensis, which has ArfT.

## MATERIALS AND METHODS

### Bacterial culture.

Bacterial strains are listed in Table S1 in the supplemental material. E. coli DH10B was used for routine cloning procedures and was grown in Luria-Bertani (LB) broth (10% Bacto tryptone, 5% yeast extract, 10% NaCl [pH 7.5]) or on LB agar supplemented with ampicillin (100 μg/ml) or kanamycin (30 μg/ml) where appropriate. F. tularensis was grown in Chamberlain’s defined medium (CDM) (40) adjusted to pH 6.2 at 37°C with shaking or on chocolate agar plates (Mueller-Hinton agar supplemented with 1% bovine hemoglobin [Remel, USA] and 1% Isovitalex X Enrichment [Becton, Dickinson, France]) at 37°C in a humidified incubator with 5% CO_2_ for 48 to 72 h. Kanamycin (10 μg/ml), tetracycline (10 μg/ml), and sucrose (5%) were added to cultures and plates where appropriate. For growth curve experiments, F. tularensis cultures were grown in CDM overnight at 37°C and 200 rpm and back diluted to an optical density at 600 nm (OD_600_) of 0.05. Growth was monitored by performing OD_600_ readings. When indicated, 1.4 μg/ml KKL-40 was added 6 h postinoculation. (Supplemental details of the materials and methods used are presented in Text S1 in the supplemental material.)

10.1128/mBio.02436-18.1 TEXT S1 Supplemental Materials and Methods. Download Text S1, PDF file, 0.1 MB. Copyright © 2018 Goralski et al. 2018 Goralski et al. This content is distributed under the terms of the Creative Commons Attribution 4.0 International license.

### Plasmid construction.

Oligonucleotide sequences are listed in Table S3 in the supplemental material. To generate plasmids pMP812-ΔArfT and pMP812-Δ0993, 600-bp PCR products flanking the gene to be deleted were amplified using primer pair ArfT_UF and ArfT_UR and primer pair ArfT_DF and ArfT_DR for pMP812-ΔArfT and primer pair 0993_UF and 0993_UR and primer pair 0993_DF and 0993_DR for pMP812-Δ0993, digested with BamHI, and ligated together. The sequence was then reamplified as one unit with primer pair ArfT_UF and ArfT_DR and primer pair 0993_UF and 0993_DR and cloned into pMP812 using SalI and NotI restriction sites. Plasmids pArfT and pFtssrA were constructed by amplifying the coding sequences of each gene using primer pair ArfT_CF and ArfT_CR and primer pair FtssrA_CF and FtssrA_CR. The Bfr promoter (41) was amplified using primers Bfr_F and Bfr_R, ligated upstream of either the ArfT or SsrA PCR product using a BamHI restriction site, and reamplified as one unit with primer Bfr_F and either primer ArfT_CR or primer ssrA_CR. The resulting PCR product was digested with EcoRI and ligated into the pKK214-MCS_4_ plasmid (41). In order to construct plasmids pET28ArfT, pET28RF1, and pET28RF2, primer pair RF1_PF and RF1_PR, primer pair RF2_PF and RF2_PR, and primer pair ArfT_PF and ArfT_PR were used to generate PCR products of the protein coding sequence of RF1, RF2, and ArfT from F. tularensis, respectively. The PCR products were then cloned into pET28a(+) using NdeI and XhoI restriction sites for protein expression of ArfT, as well as release factor 1 (RF1) and release factor 2 (RF2) from F. tularensis.

10.1128/mBio.02436-18.5 TABLE S3 Strains, plasmids, and primers used in this study. Download TABLE S3, PDF file, 0.1 MB.. Copyright © 2018 Goralski et al. 2018 Goralski et al. This content is distributed under the terms of the Creative Commons Attribution 4.0 International license.

### Tn-seq.

Overnight cultures of wild-type F. tularensis and the *ssrA*::*LtrB-bp147* strain were grown to an OD_600_ of 0.5, washed 3 times with 500 mM sucrose, and transformed with ∼300 ng of plasmid pHimar H3. Over 50,000 colonies were pooled, and chromosomal DNA was extracted. The libraries were prepared and sequenced on an Illumina HiSeq 2000 instrument by Fasteris (Geneva, Switzerland). The data were mapped to the genome of F. tularensis subsp. *holarctica* FTNF002-00 and were analyzed in Geneious version 11.1.4 using parameters described previously (9). The frequency of transposition for each gene was quantified in both strain backgrounds. Additionally, the relative fitness of each gene in the two strains was quantified by looking at the ratio of the number of times that a sequence was recovered in the *ssrA* mutant to the number seen with the wt. Insertion ratio data were generated for each gene to determine if the genes were essential in the absence of *ssrA* (see Table S1 in the supplemental material).

### Purification of ArfT, F. tulanesis RF1, and F. tulanesis RF2.

Strains TG001, TG002, and TG003 were grown to an OD_600_ of ∼0.8, and the expression of ArfT, RF1, or RF2 was induced by the addition of isopropyl-β-d-thiogalactopyranoside (IPTG) to 1 mM. Cells were harvested by centrifugation, resuspended in native lysis buffer (50 mM sodium phosphate, 300 mM NaCl, 5 mM imidazole [pH 8.0]), and sonicated or processed through a French press. The lysate was cleared by centrifugation at 14,000 × *g* for 10 min. Nickel-nitrilotriacetic acid (NTA) agarose (Qiagen) that had been equilibrated with lysis buffer was added to the cleared lysate, followed by incubation with gentle rocking at 4°C for 1 h. The slurry was packed in a column and washed with 10 volumes of native wash buffer (50 mM sodium phosphate, 300 mM NaCl, 20 mM imidazole [pH 8.0]). Bound protein was eluted with native elution buffer (50 mM sodium phosphate, 300 mM NaCl, 250 mM imidizole [pH 8.0]) and visualized by SDS-PAGE. Fractions containing 6×His-protein were dialyzed against RF or ArfT storage buffer (50 mM HEPES, 300 mM NaCl [pH 7.5] for FTA_0865, 50 mM Tris-HCl, 300 mM NaCl [pH 7.0] for RF1 and RF2). The 6×His tag was removed from RF1 by use of a Thrombin CleanCleave kit (Sigma-Aldrich) following the manufacturer’s instructions. The cleaved RF1 protein solution was loaded with NTA agarose, incubated with gentle rocking at 4°C for 1 h. The slurry was packed into a column, and the flowthrough containing RF1 was collected. RF2 was dialyzed against buffer A (50 mM Tris-HCl, 100 mM NaCl [pH 7.0]) and purified on a MonoQ column using an AKTA purifier (GE Healthcare Life Sciences). Proteins were visualized by SDS-PAGE and dialyzed into RF storage buffer.

### *In vitro* translation and peptidyl hydrolysis assays.

ArfT peptidyl hydrolysis activity was assessed using a previously described assay (9). Briefly, nonstop DHFR was PCR amplified with primers HAF_T7 and UTR_DHFR_FL, added to the PURExpress ΔRF system (New England Biolabs) A and B reaction mixtures, and incubated for 1 h at 37°C. Where indicated, ArfT was added to a final concentration of 25 μg/ml and E. coli or F. tularensis LVS RFs were added to a final concentration of 500 μg/ml, and the reaction mixtures were incubated for 1 h at 37°C. Total protein was precipitated by addition of cold acetone, resuspended in sample loading buffer (5 mM sodium bisulfite, 50 mM MOPS [morpholinepropanesulfonic acid], 50 mM Tris base, 1 µM EDTA, 0.1% SDS, 5% glycerol, 0.01% xylene cyanol, 0.01% bromophenol blue), and resolved on a Bis-Tris gel using MOPS running buffer.

### Genetic deletions.

Targeted, markerless in-frame deletions were generated for both *FTA_0865* and *FTA_0993* with a two-step allelic exchange system designed for F. tularensis using the pMP812 *sacB* suicide vector (32). F. tularensis strains were transformed with either pMP812-ArfT or pMP812-0993, and primary recombinants were selected on kanamycin after incubation at 37°C in a humidified incubator with 5% CO_2_ for 48 to 72 h. Primary recombinants were grown overnight without selection and plated on 5% sucrose to select for secondary recombinants. Secondary recombinants were confirmed by replica plating on chocolate agar containing kanamycin and on chocolate agar without selection. Genetic deletions were confirmed via PCR using primers ArfT_KOF and ArfT_KOR and primers 0993_KOF and 0993_KOR.

## References

[1] Keiler KC, Feaga HA 2014 Resolving nonstop translation complexes is a matter of life or death. J Bacteriol 196:2123–2130. doi:10.1128/JB.01490-14. 24706739 PMC4054194

[2] Keiler KC 2015 Mechanisms of ribosome rescue in bacteria. Nat Rev Microbiol 13:285–297. doi:10.1038/nrmicro3438. 25874843

[3] Ito K, Chadani Y, Nakamori K, Chiba S, Akiyama Y, Abo T 2011 Nascentome analysis uncovers futile protein synthesis in Escherichia coli. PLoS One 6:e28413. doi:10.1371/journal.pone.0028413. 22162769 PMC3230602

[4] Ito K, Uno M, Nakamura Y 2000 A tripeptide “anticodon” deciphers stop codons in messenger RNA. Nature 403:680–684. doi:10.1038/35001115. 10688208

[5] Korostelev AA 2011 Structural aspects of translation termination on the ribosome. RNA 17:1409–1421. doi:10.1261/rna.2733411. 21700725 PMC3153966

[6] Keiler KC, Waller PR, Sauer RT 1996 Role of a peptide tagging system in degradation of proteins synthesized from damaged messenger RNA. Science 271:990–993. doi:10.1126/science.271.5251.990. 8584937

[7] Karzai AW, Susskind MM, Sauer RT 1999 SmpB, a unique RNA-binding protein essential for the peptide-tagging activity of SsrA (tmRNA). EMBO J 18:3793–3799. doi:10.1093/emboj/18.13.3793. 10393194 PMC1171456

[8] Hudson CM, Lau BY, Williams KP 2014 Ends of the line for tmRNA–SmpB. Front Microbiol 5:421. doi:10.3389/fmicb.2014.00421. 25165464 PMC4131195

[9] Feaga HA, Viollier PH, Keiler KC 2014 Release of nonstop ribosomes is essential. mBio 5:1916-14. doi:10.1128/mBio.01916-14. PMC4235212 25389176

[10] Chadani Y, Ono K, Ozawa S, Takahashi Y, Takai K, Nanamiya H, Tozawa Y, Kutsukake K, Abo T 2010 Ribosome rescue by Escherichia coli ArfA (YhdL) in the absence of trans-translation system. Mol Microbiol 78:796–808. doi:10.1111/j.1365-2958.2010.07375.x. 21062370

[11] Chadani Y, Ito K, Kutsukake K, Abo T 2012 ArfA recruits release factor 2 to rescue stalled ribosomes by peptidyl-tRNA hydrolysis in Escherichia coli. Mol Microbiol 86:37–50. doi:10.1111/j.1365-2958.2012.08190.x. 22857598

[12] Shimizu Y 2012 ArfA recruits RF2 into stalled ribosomes. J Mol Biol 423:624–631. doi:10.1016/j.jmb.2012.08.007. 22922063

[13] James NR, Brown A, Gordiyenko Y, Ramakrishnan V 2016 Translation termination without a stop codon. Science 354:1437–1440. doi:10.1126/science.aai9127. 27934701 PMC5351859

[14] Zeng F, Chen y, Remis J, Shekhar M, Phillips JC, Tajkhorshid E, Jin H 2017 Structural basis of co-translational quality control by ArfA and RF2 binding to ribosome. Nature 541:554–557. doi:10.1038/nature21053. 28077875 PMC5679781

[15] Huter P, Muller C, Beckert B, Arenz S, Berninghausen O, Beckmann R, Wilson DN 2017 Structural basis for ArfA-RF2-mediated translation termination on mRNAs lacking stop codons. Nature 541:546–549. doi:10.1038/nature20821. 27906161

[16] Ma C, Kurita D, Li N, Chen Y, Himeno H, Gao N 2017 Mechanistic insights into the alternative translation termination by ArfA and RF2. Nature 541:550–553. doi:10.1038/nature20822. 27906160

[17] Chadani Y, Ono K, Kutsukake K, Abo T 2011 Escherichia coli YaeJ protein mediates a novel ribosome-rescue pathway distinct from SsrA- and ArfA-mediated pathways. Mol Microbiol 80:772–785. doi:10.1111/j.1365-2958.2011.07607.x. 21418110

[18] Handa Y, Inaho N, Nameki N 2011 YaeJ is a novel ribosome-associated protein in Escherichia coli that can hydrolyze peptidyl-tRNA on stalled ribosomes. Nucleic Acids Res 39:1739–1748. doi:10.1093/nar/gkq1097. 21051357 PMC3061065

[19] Gagnon MG, Seetharaman SV, Bulkley D, Steitz TA 2012 Structural basis for the rescue of stalled ribosomes: structure of YaeJ bound to the ribosome. Science 335:1307–1372. 10.1126/science.1217443 PMC3377438 22422986

[20] Kogure H, Handa Y, Nagata M, Kanai N, Güntert P, Kubota K, Nameki N 2014 Identification of residues required for stalled-ribosome rescue in the codon—independent release factor YaeJ. Nucleic Acids Res 42:3152–3163. doi:10.1093/nar/gkt1280. 24322300 PMC3950681

[21] Svetlanov A, Puri N, Mena P, Koller A, Karzai AW 2012 *Francisella tularensis* tmRNA system mutants are vulnerable to stress, avirulent in mice, and provide effective immune protections. Mol Microbiol 85:122–141. doi:10.1111/j.1365-2958.2012.08093.x. 22571636 PMC3395464

[22] Carvalho C, Lopes De Carvalho I, Zé-Zé L, Núncio MS, Duarte EL 2014 Tularaemia: a challenging zoonosis. Comp Immunol Microbiol Infect Dis 37:85–89. doi:10.1016/j.cimid.2014.01.002. 24480622 PMC7124367

[23] Sjöstedt A 2007 Tularemia: history, epidemiology, pathogen physiology, and clinical manifestations. Ann N Y Acad Sci 1105:1–29. doi:10.1196/annals.1409.009. 17395726

[24] Oyston PC 2008 *Francisella tularensis*: unravelling the secrets of an intracellular pathogen. J Med Microbiol 57:921–930. doi:10.1099/jmm.0.2008/000653-0. 18628490

[25] Bosio CM, Bielefeldt-Ohmann H, Belisle JT 2007 Active suppression of the pulmonary immune response by *Francisella tularensis* Schu4. J Immunol 178:4538–4547. doi:10.4049/jimmunol.178.7.4538. 17372012

[26] Tärnvik A, Berglund L 2003 Tularaemia. Eur Respir J 21:361–373. doi:10.1183/09031936.03.00088903. 12608453

[27] Center for Disease Control and Prevention (CDC). 2013 Tularemia—United States, 2001–2010. MMWR Morb Mortal Wkly Rep 62:963–966. 24280916 PMC4585636

[28] Cowley SC, Elkins KL 2011 Immunity to Francisella. Front Microbiol 2:26. doi:10.3389/fmicb.2011.00026. 21687418 PMC3109299

[29] Maier TM, Pechous R, Casey M, Zahrt TC, Frank DW 2006 In vivo Himar1-based transposon mutagenesis of Francisella tularensis. Appl Environ Microbiol 72:1878–1885. doi:10.1128/AEM.72.3.1878-1885.2006. 16517634 PMC1393221

[30] Maier TM, Casey MS, Becker RH, Dorsey CW, Glass EM, Maltsev N, Zahrt TC, Frank DW 2007 Identification of Francisella tularensis Himar1-based transposon mutants defective for replication in macrophages. Infect Immun 75:5376–5389. doi:10.1128/IAI.00238-07. 17682043 PMC2168294

[31] Madden TL, Tatusov RL, Zhang J 1996 Applications of network BLAST server. Methods Enzymol 266:131–141. doi:10.1016/S0076-6879(96)66011-X. 8743682

[32] LoVullo ED, Molins-Schneekloth CR, Schweizer HP, Pavelka MS 2009 Single-copy chromosomal integration systems for Francisella tularensis. Microbiology 155:1152–1163. doi:10.1099/mic.0.022491-0. 19332817 PMC2895234

[33] Goralski TDP, Dewan KK, Alumasa JN, Avanzato V, Place DE, Markley RL, Katkere B, Rabadi SM, Bakshi CS, Keiler KC, Kirimanjeswara GS 2016 Inhibitors of ribosome rescue arrest growth of *Francisella tularensis* at all stages of intracellular replication. Antimicrob Agents Chemother 60:3276–3282. doi:10.1128/AAC.03089-15. 26953190 PMC4879415

[34] Ramadoss NS, Alumasa JN, Cheng L, Wang Y, Li S, Chambers BS, Chang H, Chatterjee AK, Brinker A, Engels IH, Keiler KC 2013 Small molecule inhibitors of *trans*-translation have broad-spectrum antibiotic activity. Proc Natl Acad Sci U S A 110:10282–10287. doi:10.1073/pnas.1302816110. 23733947 PMC3690859

[35] Alumasa JN, Manzanillo PS, Peterson ND, Lundrigan T, Baughn AD, Cox JS, Keiler KC 2017 Ribosome rescue inhibitors kill actively growing and nonreplicating persister Mycobacterium tuberculosis cells. ACS Infect Dis 3:634–644. doi:10.1021/acsinfecdis.7b00028. 28762275 PMC5594445

[36] Huter P, Müller C, Arenz S, Beckert B, Wilson D 2017 Structural basis for ribosome rescue in bacteria. Trends Biochem Sci 42:669–680. doi:10.1016/j.tibs.2017.05.009. 28629612

[37] Kurita D, Chadani Y, Muto A, Abo T, Himeno H 2014 ArfA recognizes the lack of mRNA in the mRNA channel after RF2 binding for ribosome rescue. Nucleic Acids Res 42:13339–13352. doi:10.1093/nar/gku1069. 25355516 PMC4245945

[38] Chadani Y, Matsumoto E, Aso H, Wada T, Kutsukake K, Sutou S, Abo T 2011 *Trans*-translation-mediated tight regulation of the expression of the alternative ribosome-rescue factor ArfA in *Escherichia coli*. Genes Genet Syst 86:151–163. doi:10.1266/ggs.86.151. 21952205

[39] Garza-Sanchez F, Schaub RE, Janssen BD, Hayes CS 2011 tmRNA regulates synthesis of the ArfA ribosome rescue factor. Mol Microbiol 80:1204–1219. doi:10.1111/j.1365-2958.2011.07638.x. 21435036 PMC3103599

[40] Chamberlain RE 1965 Evaluation of live tularemia vaccine prepared in a chemically defined medium. Appl Microbiol 13:232–235. 14325885 10.1128/am.13.2.232-235.1965 PMC1058227

[41] Williamson DR, Dewan KK, Patel T, Wastella CM, Ning G, Kirimanjeswara GS 14 2 2018 A single mechanosensitive channel protects *Francisella tularensis* subsp. *holarctica* from hypoosmotic shock and promotes survival in the aquatic environment. Appl Environ Microbiol doi:10.1128/AEM.02203-17. PMC5812925 29269496

