# Supplementary material for: A New Mechanism for Ribosome Rescue Can Recruit RF1 or RF2 to Nonstop Ribosomes
Source: mBio. 2018 Dec 18;9(6):e02436-18. doi: 10.1128/mBio.02436-18 (PMC6299226; doi:10.1128/mBio.02436-18)
Supplement: TABLE S1 [file mbo006184222st1.pdf]

Table S1: Quantifiaction of transposon insertions.

Gene *	Start	End	Strand	No. of Insertions in Indicated Background Wild Type	ssrA::LtrB-A147	Log 10-1 Ratio (A147/wt)
pdpCII	1102475	1106401	-	1231	775	-0.20
pdpC1	107169	111095	-	1248	739	-0.23
pdpBII	1113624	1116845	-	1113	585	-0.28
carB	27189	30413	-	833	583	-0.15
pdpB1	118318	121539	-	1106	567	-0.29
FTA_0052	44062	47235	-	944	503	-0.27
FTA_1400	1258424	1263292	-	1123	493	-0.36
FTA_0826	768970	771083	+	622	492	-0.10
FTA_0176	166124	168218	-	715	492	-0.16
FTA_1554	1390407	1392590	+	711	451	-0.20
FTA_0103	89103	91622	-	727	413	-0.25
FTA_1605	1445148	1447412	-	677	399	-0.23
pdpA1	121604	124006	-	770	385	-0.30
pdpAII	1116910	1119312	-	803	380	-0.32
FTA_1024	941593	944222	+	604	375	-0.21
FTA_1727	1560675	1562898	+	667	368	-0.26
hrpA	574516	578790	+	949	361	-0.42
pulA	462228	465380	-	800	355	-0.35
FTA_0373	330773	331593	-	392	354	-0.04
ppdK	133958	136546	-	665	354	-0.27
FTA_1718	1553556	1555868	+	773	353	-0.34
kdpA	1811348	1813009	-	587	348	-0.23
FTA_1900	1721081	1723864	-	736	342	-0.33
uvrA	1392656	1395418	-	754	340	-0.35
FTA_0088	76540	77309	-	365	338	-0.03
FTA_0073	63522	65741	-	633	333	-0.28
mfd	891419	894784	-	746	333	-0.35
pepN	1877932	1880466	-	779	332	-0.37
FTA_0102	86660	88792	-	669	328	-0.31
FTA_1769	1601223	1604276	+	830	328	-0.40
rnr	539428	541539	+	518	327	-0.20
FTA_1865	1688632	1690524	-	666	323	-0.31
FTA_0130	115775	117379	-	552	321	-0.24
FTA_1231	1111081	1112685	-	556	320	-0.24
FTA_2000	1818887	1819656	+	366	318	-0.06
mutS	278528	281002	-	680	311	-0.34
FTA_0887	820855	822267	-	385	306	-0.10
FTA_1045	962604	963699	-	373	305	-0.09
FTA_0826	770269	771089	-	312	304	-0.01
FTA_1393	1254228	1255163	-	461	303	-0.18
FTA_1778	1610598	1611418	+	311	301	-0.01

FTA_0618	564032	566668	-	579	301	-0.28
FTA_0560	510296	513109	+	680	300	-0.36
FTA_0182	169306	171987	-	689	294	-0.37
FTA_1628	1469755	1470575	+	299	293	-0.01
FTA_0439	384699	385478	+	319	293	-0.04
FTA_1045	962598	963418	+	300	292	-0.01
FTA_0176	167154	167974	-	315	292	-0.03
FTA_1325	1195199	1196680	+	482	292	-0.22
FTA_1391	1253152	1253972	+	308	291	-0.02
FTA_1716	1552451	1553271	+	304	289	-0.02
FTA_1229	1109049	1110419	-	483	289	-0.22
FTA_1922	1744790	1745610	-	294	288	-0.01
FTA_0114	100077	100897	+	306	288	-0.03
FTA_1861	1687774	1688594	+	319	288	-0.04
FTA_0465	410994	411860	+	348	288	-0.08
FTA_1261	1143787	1144607	+	308	287	-0.03
FTA_1080	983654	984660	+	360	287	-0.10
FTA_2032	1851937	1852757	-	313	286	-0.04
FTA_0128	113743	115113	-	518	286	-0.26
FTA_1189	1072697	1073517	-	299	285	-0.02
FTA_1450	1300420	1301240	-	307	285	-0.03
FTA_1696	1533929	1534749	-	320	285	-0.05
FTA_1455	1305845	1308316	+	693	285	-0.39
FTA_0719	671332	672152	-	312	284	-0.04
FTA_2057	1869772	1871391	-	431	284	-0.18
FTA_1364	1230814	1231634	-	283	282	0.00
FTA_0028	21134	21954	+	304	282	-0.03
FTA_0279	248651	249586	-	461	282	-0.21
FTA_0277	247575	248395	+	309	281	-0.04
FTA_2054	1868057	1868877	+	310	281	-0.04
FTA_1532	1371548	1372327	+	328	281	-0.07
FTA_1785	1616085	1616905	+	305	280	-0.04
FTA_1426	1281363	1282142	+	326	280	-0.07
FTA_0072	62319	63395	+	443	280	-0.20
FTA_1668	1506040	1507389	+	437	279	-0.19
FTA_1738	1569154	1569974	+	311	278	-0.05
FTA_0439	385327	386088	-	279	277	0.00
FTA_0061	54172	54992	-	310	277	-0.05
FTA_0837	779414	782074	+	604	277	-0.34
FTA_0138	130410	131230	+	306	276	-0.04
FTA_0773	724441	725261	+	288	275	-0.02
FTA_0004	3092	3912	-	299	275	-0.04
FTA_1372	1237038	1237858	+	294	273	-0.03
FTA_1313	1187988	1188808	-	298	273	-0.04
FTA_0199	184080	184900	+	306	273	-0.05

FTA_0360	320752	321572	+	317	273	-0.06
FTA_0166	158768	159588	+	288	272	-0.02
FTA_0529	490435	491255	-	304	272	-0.05
FTA_0814	763654	765931	+	604	272	-0.35
FTA_0664	621073	621893	-	274	270	-0.01
FTA_1532	1372171	1372937	-	288	270	-0.03
FTA_0712	667123	667943	-	300	270	-0.05
FTA_1215	1095383	1096203	+	309	270	-0.06
FTA_0740	690639	692323	+	379	270	-0.15
FTA_1077	980437	981257	+	296	269	-0.04
FTA_1721	1555929	1556749	-	328	269	-0.09
FTA_1020	939036	939856	-	316	268	-0.07
FTA_1814	1640199	1641019	+	302	267	-0.05
fadE	566801	569002	-	557	267	-0.32
FTA_1549	1386342	1387162	-	289	266	-0.04
glgC	469366	470577	+	406	266	-0.18
FTA_0332	301314	302134	+	301	265	-0.06
FTA_0018	12982	15018	+	419	265	-0.20
FTA_1589	1433115	1433935	-	297	263	-0.05
FTA_0145	140190	141010	-	300	262	-0.06
FTA_1820	1645291	1646111	-	299	261	-0.06
FTA_0321	288159	289268	+	351	261	-0.13
FTA_1995	1814551	1815320	-	275	260	-0.02
FTA_0020	15990	16810	-	302	260	-0.07
FTA_0463	409974	410743	+	264	259	-0.01
FTA_0641	597629	598449	+	295	259	-0.06
FTA_1488	1330447	1331267	-	281	258	-0.04
FTA_1746	1575901	1577303	-	437	256	-0.23
glgP	472221	474434	+	616	256	-0.38
FTA_0502	454605	456686	+	535	255	-0.32
FTA_0108	95122	96567	-	466	254	-0.26
FTA_1574	1410206	1413181	-	618	254	-0.39
FTA_1048	963763	965193	-	411	253	-0.21
katG	1430121	1432286	-	525	252	-0.32
FTA_1172	1057761	1058498	+	278	251	-0.04
FTA_0447	391271	392653	+	372	251	-0.17
FTA_0281	250482	251248	-	262	248	-0.02
FTA_0537	495882	496702	-	281	248	-0.05
FTA_0285	253586	254406	-	286	247	-0.06
ggt	756970	758715	+	435	243	-0.25
FTA_1990	1809361	1811278	-	549	243	-0.35
FTA_1731	1564146	1565144	-	275	242	-0.06
FTA_0008	4710	6050	-	421	242	-0.24
FTA_0794	745457	747148	+	434	242	-0.25
FTA_0828	771378	773066	-	406	241	-0.23

FTA_1710	1545907	1547688	-	431	241	-0.25
FTA_1944	1767177	1768646	+	432	237	-0.26
FTA_1661	1499187	1500929	-	445	237	-0.27
FTA_1735	1567114	1568556	+	536	237	-0.35
FTA_1753	1580347	1582509	-	584	237	-0.39
leuC	1817190	1818533	-	463	235	-0.29
gcvP1	458419	459726	+	379	234	-0.21
FTA_0160	152783	154261	+	504	233	-0.34
FTA_2024	1843653	1845608	-	644	233	-0.44
FTA_1128	1023613	1025096	+	359	232	-0.19
FTA_1750	1577432	1579339	-	520	232	-0.35
FTA_1292	1171237	1172544	+	327	231	-0.15
iglB	1098468	1099952	+	457	231	-0.30
FTA_0300	267733	268869	+	364	230	-0.20
FTA_2007	1825471	1826952	+	419	230	-0.26
FTA_0959	885002	886259	+	325	227	-0.16
FTA_1014	932001	933398	-	426	227	-0.27
FTA_0696	644007	645581	+	445	227	-0.29
FTA_0303	269689	271572	+	482	227	-0.33
FTA_0364	323293	324762	+	415	226	-0.26
FTA_0044	38337	39329	-	403	225	-0.25
iglB1	103186	104646	+	477	223	-0.33
FTA_1940	1761451	1763310	+	461	222	-0.32
FTA_1029	947725	949992	+	467	222	-0.32
FTA_1413	1271372	1272890	-	394	221	-0.25
FTA_1323	1194420	1195052	+	245	220	-0.05
FTA_1994	1813545	1814300	-	299	220	-0.13
recN	357763	359352	+	494	220	-0.35
FTA_1673	1512802	1513500	-	254	219	-0.06
FTA_0049	42494	43087	-	237	217	-0.04
FTA_0655	613682	614281	-	250	217	-0.06
FTA_0609	556099	556707	-	248	216	-0.06
FTA_1657	1496232	1496858	+	264	216	-0.09
FTA_1282	1162961	1164796	-	486	215	-0.35
FTA_1600	1442308	1442898	+	230	214	-0.03
FTA_0389	340490	341119	+	260	214	-0.08
FTA_0791	742236	742826	+	274	214	-0.11
FTA_1168	1054076	1055395	+	374	214	-0.24
FTA_0647	605458	606111	-	240	213	-0.05
FTA_1752	1579433	1580023	-	243	213	-0.06
FTA_2002	1819907	1820527	+	268	212	-0.10
FTA_0413	359713	360885	-	437	212	-0.31
FTA_0423	370712	371305	-	258	211	-0.09
trpE	1886644	1888128	-	380	211	-0.26
FTA_0652	610213	611537	+	407	211	-0.29

FTA_2058	1871619	1872836	+	477	211	-0.35
FTA_0087	75696	76289	-	236	210	-0.05
FTA_1986	1806004	1807954	-	586	210	-0.45
FTA_1454	1303170	1305312	-	653	210	-0.49
FTA_1585	1426538	1427176	-	259	208	-0.10
FTA_1551	1387500	1388096	+	260	208	-0.10
FTA_0222	205371	206936	+	434	207	-0.32
FTA_1423	1279255	1280645	+	443	207	-0.33
FTA_1495	1335950	1337605	+	443	207	-0.33
FTA_0913	842397	842996	+	252	206	-0.09
FTA_0316	283702	285603	+	542	206	-0.42
FTA_1472	1315298	1315921	-	243	205	-0.07
FTA_1957	1778713	1779309	-	255	205	-0.09
FTA_0038	32061	33056	+	314	205	-0.19
FTA_1723	1556871	1557845	-	361	205	-0.25
FTA_1145	1036017	1036577	-	216	204	-0.02
FTA_0868	805292	805881	+	237	204	-0.07
FTA_2142	220215	220838	-	238	204	-0.07
FTA_0068	60235	61059	-	320	204	-0.20
glgB	465474	467336	-	348	204	-0.23
FTA_1833	1657087	1658550	+	449	204	-0.34
feoB	137418	139601	-	563	204	-0.44
FTA_0287	256466	257095	+	251	203	-0.09
FTA_1528	1368325	1368966	+	258	203	-0.10
uvrB	736977	738923	+	465	203	-0.36
FTA_1152	1039327	1041221	+	485	203	-0.38
FTA_0494	444488	445123	-	251	202	-0.09
FTA_0161	154427	156241	+	388	202	-0.28
FTA_1827	1650651	1651961	+	404	202	-0.30
polA	1592527	1595160	-	441	201	-0.34
FTA_1376	1238999	1239604	+	235	199	-0.07
FTA_0920	850820	851421	-	236	199	-0.07
FTA_1554	1391414	1392019	+	237	199	-0.08
FTA_0700	648483	650009	+	307	199	-0.19
FTA_0025	19311	20432	-	348	199	-0.24
FTA_0237	221341	222573	+	428	199	-0.33
FTA_1638	1478352	1480205	-	504	199	-0.40
FTA_2125	1121522	1124413	+	300	198	-0.18
galE	1360476	1361435	-	308	198	-0.19
FTA_1798	1624286	1625905	-	463	198	-0.37
FTA_1593	1435077	1436411	-	330	197	-0.22
FTA_1706	1543878	1545277	+	419	197	-0.33
FTA_1113	1010489	1012439	-	421	197	-0.33
rlmL	1226600	1228693	-	471	197	-0.38
FTA_1163	1047602	1049427	+	479	197	-0.39

FTA_0625	579384	579944	+	221	196	-0.05
FTA_1986	1807676	1808626	-	293	196	-0.17
FTA_1355	1221203	1222810	+	438	196	-0.35
FTA_0031	22961	24124	-	371	195	-0.28
FTA_0602	549233	549793	-	216	194	-0.05
FTA_0404	352252	353586	-	336	193	-0.24
gcvP2	459792	461177	+	370	193	-0.28
iglD1	105373	106509	+	391	193	-0.31
FTA_0669	623221	624667	+	416	193	-0.33
FTA_1777	1609640	1610260	-	259	192	-0.13
FTA_1652	1490751	1491857	-	335	192	-0.24
FTA_0045	39376	40281	-	304	191	-0.20
FTA_2122	126216	129107	+	306	190	-0.21
FTA_1975	1795412	1796542	-	315	190	-0.22
FTA_0553	504259	506238	+	481	190	-0.40
FTA_2123	419203	422094	+	296	188	-0.20
FTA_0527	487944	489533	+	410	188	-0.34
FTA_1587	1427485	1428648	+	441	188	-0.37
radA	345634	346944	-	290	185	-0.20
nhaA	79907	80998	+	353	185	-0.28
FTA_0736	686626	687666	-	309	184	-0.23
FTA_1913	1734508	1735746	-	362	184	-0.29
FTA_1217	1096264	1097076	-	267	183	-0.16
FTA_1711	1547782	1548948	-	293	183	-0.20
FTA_2027	1847585	1848974	+	409	183	-0.35
FTA_0845	786244	787956	+	435	183	-0.38
parC	437486	439651	+	469	183	-0.41
FTA_0606	552062	553432	+	343	182	-0.28
FTA_1058	971343	972737	+	382	182	-0.32
iglDII	1100670	1101815	+	388	182	-0.33
FTA_0290	259237	260556	+	467	182	-0.41
FTA_0800	750238	751433	+	330	181	-0.26
dprA	309042	310087	-	395	181	-0.34
FTA_0466	412569	413732	-	411	181	-0.36
FTA_1331	1202173	1203370	+	415	181	-0.36
FTA_0410	355397	356527	-	328	180	-0.26
FTA_0370	329159	330254	+	315	179	-0.25
recC	650146	653325	+	309	178	-0.24
FTA_1744	1573723	1574865	+	381	177	-0.33
FTA_0116	100958	101770	-	254	176	-0.16
FTA_1504	1344479	1345636	-	308	176	-0.24
uvrC	1373516	1375294	-	350	175	-0.30
glpK	1570031	1571479	-	396	175	-0.35
FTA_1564	1400507	1401886	-	408	175	-0.37
FTA_1197	1077696	1078844	-	344	174	-0.30

FTA_1584	1424955	1426439	-	443	174	-0.41
iglCII	1100023	1100598	+	219	173	-0.10
FTA_1158	1045713	1046739	-	262	172	-0.18
FTA_1648	1487484	1488344	+	268	172	-0.19
FTA_1695	1531884	1533584	+	453	172	-0.42
asd	481661	482641	-	266	171	-0.19
FTA_1186	1070423	1071637	-	285	171	-0.22
FTA_0962	886893	887982	+	305	171	-0.25
FTA_0172	162466	163881	+	413	171	-0.38
FTA_1335	1203987	1205690	-	461	171	-0.43
FTA_0792	743000	744307	+	381	170	-0.35
FTA_1626	1466668	1468707	-	397	170	-0.37
FTA_0620	569079	570707	-	437	170	-0.41
FTA_1468	1312548	1313632	-	349	169	-0.31
ackA	11818	12912	+	255	168	-0.18
FTA_1642	1483436	1484402	+	282	168	-0.22
iglC1	104717	105292	+	233	167	-0.14
FTA_1482	1324853	1326148	-	246	167	-0.17
pilB	810263	811984	-	388	167	-0.37
FTA_0742	692448	693845	-	377	166	-0.36
FTA_0975	899442	900853	+	411	166	-0.39
FTA_1516	1357564	1359186	-	423	166	-0.41
mviN	1242578	1244059	-	427	166	-0.41
FTA_1184	1068841	1069872	+	272	165	-0.22
FTA_1828	1652021	1653142	+	330	165	-0.30
FTA_1514	1356264	1357490	-	363	165	-0.34
FTA_1768	1599850	1601163	+	372	165	-0.35
FTA_1582	1423186	1424376	-	428	165	-0.41
FTA_0339	305579	307105	-	431	165	-0.42
FTA_1536	1375463	1376494	+	234	164	-0.15
FTA_1866	1690588	1691481	-	323	164	-0.29
FTA_1943	1765901	1767031	+	356	164	-0.34
FTA_1910	1731210	1732454	+	360	164	-0.34
FTA_0108	96563	97897	-	364	164	-0.35
FTA_1977	1798259	1799774	-	416	164	-0.40
FTA_1645	1486036	1487420	+	436	164	-0.42
FTA_1116	1012502	1013905	-	457	164	-0.45
FTA_0367	326709	327405	+	245	163	-0.18
FTA_1541	1379428	1380489	+	286	163	-0.24
rimO	318952	320187	+	319	163	-0.29
FTA_1271	1152803	1154173	-	325	163	-0.30
FTA_0917	847046	848389	+	341	163	-0.32
FTA_0773	725281	727143	+	437	163	-0.43
recB	655544	659137	+	268	161	-0.22
FTA_1510	1351869	1352822	-	282	161	-0.24

FTA_1713	1550437	1551519	+	309	161	-0.28
FTA_2008	1827021	1827758	+	236	160	-0.17
FTA_2046	1861567	1862721	-	403	160	-0.40
FTA_0309	275357	276508	-	428	160	-0.43
FTA_1448	1298931	1300263	+	418	159	-0.42
FTA_2035	1852879	1853304	-	182	158	-0.06
FTA_0227	212394	213320	+	336	158	-0.33
cyoB	193229	195106	+	430	158	-0.43
FTA_2043	1859112	1859915	-	274	157	-0.24
FTA_1012	930939	931861	-	297	157	-0.28
FTA_1759	1588581	1589720	-	319	157	-0.31
FTA_0885	819664	820751	-	325	157	-0.32
FTA_1154	1042765	1043866	-	345	157	-0.34
FTA_1770	1604468	1605688	+	358	157	-0.36
putA	790998	795002	+	530	157	-0.53
recD	659190	660971	+	263	156	-0.23
FTA_0227	211552	212379	+	283	156	-0.26
FTA_1278	1158578	1159708	-	317	156	-0.31
FTA_1502	1342777	1343928	-	322	156	-0.31
glgA	470647	472056	+	266	155	-0.23
FTA_1982	1802796	1803932	-	307	155	-0.30
FTA_1505	1345772	1346968	-	415	155	-0.43
FTA_0058	51238	52410	-	438	155	-0.45
recJ	1508651	1510333	+	370	154	-0.38
typA	759718	761475	-	314	153	-0.31
FTA_1812	1638923	1640145	-	356	153	-0.37
FTA_0362	321920	323017	+	255	152	-0.22
FTA_1517	1359243	1360412	-	293	152	-0.29
FTA_1952	1773926	1774939	-	303	152	-0.30
FTA_0294	262417	263775	+	430	152	-0.45
FTA_0608	554492	555826	+	370	151	-0.39
FTA_1460	1308997	1310430	-	395	150	-0.42
FTA_0297	264827	265483	-	255	149	-0.23
FTA_1272	1154325	1155692	-	332	149	-0.35
FTA_2142	219468	220223	+	235	148	-0.20
FTA_1795	1622973	1624215	-	332	148	-0.35
FTA_0322	289725	290963	-	401	148	-0.43
FTA_1699	1534968	1535864	-	223	147	-0.18
FTA_0386	338540	339535	+	284	147	-0.29
FTA_0409	354251	355288	+	299	147	-0.31
FTA_0790	740705	741922	+	300	147	-0.31
htpG	251470	253296	+	315	147	-0.33
FTA_0907	837618	838595	+	233	146	-0.20
FTA_0795	747258	748358	-	291	146	-0.30
FTA_1567	1404263	1405456	+	314	146	-0.33

FTA_1369	1234413	1235680	-	346	146	-0.37
FTA_0389	341118	342556	-	388	146	-0.42
rep	1061149	1063104	+	476	146	-0.51
FTA_1394	1255580	1256035	+	198	145	-0.14
FTA_0699	647434	648246	-	235	145	-0.21
FTA_0211	197123	198098	-	264	145	-0.26
FTA_1598	1440975	1442030	+	300	145	-0.32
FTA_1594	1436487	1437743	-	335	145	-0.36
FTA_0205	190468	191718	-	371	145	-0.41
FTA_1095	994083	995434	+	428	145	-0.47
FTA_1572	1408789	1409532	+	212	144	-0.17
FTA_1511	1352885	1353832	-	268	144	-0.27
FTA_0986	910445	911521	-	307	144	-0.33
xseA	748571	749896	+	340	144	-0.37
purT	85446	86546	-	373	144	-0.41
FTA_0125	111934	113025	-	296	143	-0.32
carA	30478	31584	-	308	143	-0.33
FTA_1366	1231788	1232846	+	236	142	-0.22
FTA_1226	1107240	1108331	-	308	142	-0.34
FTA_1250	1133042	1134253	+	401	142	-0.45
FTA_0730	681220	682647	-	420	142	-0.47
FTA_1964	1782956	1784083	-	279	140	-0.30
FTA_1870	1694480	1695514	+	308	140	-0.34
FTA_0607	553503	554426	+	322	140	-0.36
FTA_1658	1496929	1498035	+	362	140	-0.41
FTA_0876	809019	810188	-	306	139	-0.34
FTA_0994	916646	917832	+	323	139	-0.37
FTA_0472	422641	423267	+	182	138	-0.12
FTA_0992	914332	915447	+	308	138	-0.35
FTA_1712	1549091	1550074	-	309	138	-0.35
FTA_0584	534685	535715	+	336	138	-0.39
lysA	258056	259165	+	361	138	-0.42
FTA_1666	1503707	1504462	+	212	137	-0.19
pyrB	26127	26987	-	244	137	-0.25
FTA_1974	1794041	1795312	+	328	137	-0.38
FTA_1416	1274172	1275600	-	333	137	-0.39
FTA_0677	629045	629770	-	192	136	-0.15
FTA_0546	500556	501299	-	193	136	-0.15
FTA_1793	1621768	1622511	-	246	136	-0.26
FTA_0727	676912	678381	-	254	136	-0.27
murQ	285660	286484	+	282	136	-0.32
FTA_1741	1571607	1572758	-	287	136	-0.32
FTA_1775	1608019	1608972	-	295	136	-0.34
FTA_0919	849327	850724	-	322	136	-0.37
FTA_0929	859113	859916	-	223	135	-0.22

FTA_0540	497012	497632	+	231	135	-0.23
FTA_1312	1186688	1187740	+	240	135	-0.25
FTA_1270	1151646	1152575	-	260	135	-0.28
FTA_1026	944297	945394	+	286	135	-0.33
FTA_0060	52489	53853	-	382	135	-0.45
FTA_1766	1598224	1599246	+	236	134	-0.25
FTA_1481	1323427	1324761	-	306	134	-0.36
FTA_0963	888309	889843	+	362	134	-0.43
FTA_0557	509051	509709	+	171	133	-0.11
pheA	1270444	1271226	-	188	133	-0.15
FTA_1181	1067147	1067890	+	204	133	-0.19
FTA_1351	1218075	1219274	-	293	133	-0.34
FTA_0200	184938	185996	-	360	133	-0.43
parE	1653292	1655115	+	368	133	-0.44
FTA_0617	562832	563959	-	229	132	-0.24
prfC	1585079	1586596	-	251	132	-0.28
FTA_1782	1613247	1614131	+	271	132	-0.31
FTA_1167	1052691	1054013	+	309	132	-0.37
FTA_0442	386535	388481	-	408	132	-0.49
FTA_0732	684412	685050	-	155	131	-0.07
FTA_0007	3963	4640	+	226	131	-0.24
FTA_0521	483000	483797	+	253	131	-0.29
FTA_0745	696103	697314	-	297	131	-0.36
trpD	1885052	1886017	-	323	131	-0.39
FTA_1953	1775121	1776482	-	401	131	-0.49
FTA_0238	223127	223990	-	235	130	-0.26
FTA_0365	325082	325954	+	254	130	-0.29
FTA_1876	1698060	1699100	-	255	130	-0.29
FTA_2051	1863980	1865107	+	265	130	-0.31
FTA_0821	767296	768314	+	237	129	-0.26
FTA_1034	952821	953810	-	243	129	-0.28
FTA_0950	879204	880355	+	273	129	-0.33
FTA_0224	207679	208101	+	155	128	-0.08
ilvC	890253	891236	+	218	128	-0.23
FTA_1787	1617278	1618069	-	243	128	-0.28
FTA_2025	1845832	1846928	+	330	128	-0.41
FTA_0597	546346	547341	-	301	127	-0.37
FTA_2131	417423	418891	+	182	126	-0.16
FTA_1134	1029735	1030265	-	187	126	-0.17
FTA_0890	824615	825297	+	211	126	-0.22
FTA_1597	1440046	1440918	+	241	126	-0.28
FTA_1156	1044193	1045254	-	249	126	-0.30
FTA_2056	1869073	1869695	-	179	125	-0.16
FTA_1291	1169767	1170948	-	323	125	-0.41
FTA_2043	1859896	1860564	-	181	124	-0.16

rsgA	1502601	1503428	+	188	124	-0.18
glpX	1627809	1628735	-	242	124	-0.29
FTA_1033	951389	952741	+	274	124	-0.34
FTA_1595	1437910	1438884	-	286	124	-0.36
FTA_0492	440976	442034	-	290	124	-0.37
FTA_0010	6937	7800	+	320	124	-0.41
FTA_2082	1888958	1890394	-	327	124	-0.42
FTA_0596	545577	546292	-	198	123	-0.21
FTA_1538	1376583	1377737	+	258	123	-0.32
FTA_0519	480794	481552	+	265	123	-0.33
FTA_1196	1076554	1077546	-	269	123	-0.34
FTA_1882	1703430	1704512	+	276	123	-0.35
FTA_0997	917899	919126	-	323	123	-0.42
lon	873460	875724	+	379	123	-0.49
FTA_2130	124436	125904	+	183	122	-0.18
trmB	619075	619704	+	170	121	-0.15
FTA_0896	828431	829186	+	225	121	-0.27
FTA_0930	858058	859062	+	235	121	-0.29
prmA	945677	946462	+	241	121	-0.30
FTA_0382	335805	336818	-	263	121	-0.34
FTA_0081	70452	71384	-	264	121	-0.34
FTA_0786	735527	736915	+	318	121	-0.42
FTA_0951	880426	881543	-	318	121	-0.42
FTA_0220	203712	204650	+	267	120	-0.35
FTA_1644	1484788	1485747	-	273	120	-0.36
FTA_0812	762025	763449	+	336	120	-0.45
FTA_2044	1860575	1861432	-	229	119	-0.28
FTA_0682	631942	633014	-	245	119	-0.31
FTA_0728	679421	681052	-	294	119	-0.39
FTA_0310	276592	277752	-	299	119	-0.40
lnt	862172	863602	-	307	119	-0.41
FTA_1387	1250262	1251430	+	313	119	-0.42
trxB	1495013	1495903	+	217	118	-0.26
FTA_1653	1492028	1492894	+	275	118	-0.37
FTA_2009	1828006	1828488	+	183	117	-0.19
FTA_0399	347779	348318	-	237	117	-0.31
FTA_0056	49454	50230	-	250	117	-0.33
FTA_1519	1361488	1362822	-	321	117	-0.44
FTA_0939	868196	868879	+	180	116	-0.19
FTA_2132	1119742	1121210	+	189	116	-0.21
FTA_1331	1201206	1202150	+	248	116	-0.33
FTA_0931	860405	861295	+	254	116	-0.34
FTA_1726	1559145	1560077	-	264	116	-0.36
FTA_0751	703685	704818	+	265	116	-0.36
FTA_0914	843091	844425	+	305	116	-0.42

FTA_1612	1453963	1455156	-	305	116	-0.42
fumC	216392	217723	+	351	116	-0.48
FTA_0693	642114	642812	+	149	115	-0.11
FTA_1960	1781166	1781966	-	240	115	-0.32
FTA_0047	40963	41772	+	269	115	-0.37
FTA_1085	987394	988446	+	280	115	-0.39
FTA_1969	1789599	1790842	-	281	115	-0.39
FTA_1586	1428692	1429957	-	288	115	-0.40
FTA_1213	1093523	1094942	-	318	115	-0.44
pcp	207003	207611	+	162	114	-0.15
FTA_2079	1886062	1886580	-	195	114	-0.23
FTA_1831	1655821	1656387	+	207	114	-0.26
FTA_0090	77613	78203	-	213	114	-0.27
FTA_0873	807791	808885	+	214	114	-0.27
FTA_0473	423709	424704	-	231	114	-0.31
FTA_1635	1475605	1476528	+	259	113	-0.36
FTA_0065	57655	58800	-	264	113	-0.37
FTA_1873	1696994	1697920	+	268	113	-0.38
FTA_0947	876256	877629	+	296	113	-0.42
FTA_1871	1695578	1696480	+	352	113	-0.49
FTA_0613	559132	560487	+	354	113	-0.50
FTA_1056	969418	970128	+	175	112	-0.19
FTA_1577	1416403	1417242	+	183	112	-0.21
FTA_0616	561009	562229	+	362	112	-0.51
FTA_1791	1620582	1621702	-	423	112	-0.58
FTA_0381	335065	335640	-	172	111	-0.19
FTA_1660	1498167	1498943	-	223	111	-0.30
FTA_0298	266658	267482	-	234	111	-0.32
FTA_1868	1693081	1693902	-	277	111	-0.40
FTA_0021	15093	15952	+	215	110	-0.29
rdgC	1065361	1066161	+	226	110	-0.31
FTA_1022	939984	941093	+	235	110	-0.33
FTA_1465	1311550	1312467	-	252	110	-0.36
FTA_0703	654042	655412	+	277	110	-0.40
FTA_1921	1743352	1744715	-	304	110	-0.44
FTA_1856	1682546	1683733	-	347	110	-0.50
FTA_0928	856167	857900	+	425	110	-0.59
FTA_1655	1493712	1494746	+	271	109	-0.40
FTA_2042	1858301	1858954	-	197	108	-0.26
FTA_0656	614632	615495	+	199	108	-0.27
FTA_1037	954590	955576	+	201	108	-0.27
FTA_0808	755995	756855	+	211	108	-0.29
cyoE	196130	196918	+	282	108	-0.42
FTA_1568	1405523	1406686	+	289	108	-0.43
FTA_1427	1282432	1282752	-	116	107	-0.04

FTA_0096	81848	82528	-	143	107	-0.13
FTA_1319	1191251	1191694	+	148	107	-0.14
FTA_1398	1257488	1258383	-	222	107	-0.32
FTA_0027	20528	21080	-	236	107	-0.34
FTA_0657	615564	616283	+	249	107	-0.37
FTA_0140	131579	132599	-	271	107	-0.40
FTA_0867	803793	804965	-	273	107	-0.41
FTA_0532	491512	492312	+	173	106	-0.21
FTA_0346	310147	311340	-	178	106	-0.23
FTA_1350	1217189	1218013	-	182	106	-0.23
FTA_0293	261695	262354	+	222	106	-0.32
FTA_0860	800504	801306	-	244	106	-0.36
FTA_0216	200366	201262	+	252	106	-0.38
ttg2B	499788	500492	-	273	106	-0.41
FTA_0434	379237	380052	-	179	105	-0.23
FTA_1984	1804587	1805164	-	182	105	-0.24
FTA_0528	489574	490374	+	184	105	-0.24
FTA_1290	1168311	1169375	-	221	105	-0.32
FTA_1076	981295	982290	-	263	105	-0.40
FTA_1473	1316251	1317134	-	271	105	-0.41
FTA_0576	527556	528539	+	283	105	-0.43
FTA_0764	715601	716089	-	172	104	-0.22
FTA_0127	113107	113670	-	212	104	-0.31
FTA_1836	1660198	1660989	-	220	104	-0.33
rnhB	1132486	1132989	+	121	103	-0.07
FTA_1320	1191754	1192413	+	178	103	-0.24
FTA_0450	395894	396748	+	182	103	-0.25
FTA_1273	1155895	1156969	-	256	103	-0.40
trpCF	1881135	1882436	-	264	103	-0.41
ilvE	132670	133497	-	290	103	-0.45
FTA_1263	1144808	1145490	+	189	102	-0.27
FTA_0680	630543	631292	-	205	102	-0.30
FTA_1343	1210412	1211256	+	259	102	-0.40
FTA_1111	1009126	1010316	+	295	102	-0.46
FTA_1942	1764632	1765825	+	329	102	-0.51
FTA_0909	839699	840244	-	156	101	-0.19
FTA_1003	923311	924059	-	197	101	-0.29
FTA_1673	1512097	1512904	-	201	101	-0.30
FTA_1173	1058609	1059226	-	210	101	-0.32
FTA_2152	1875310	1876001	+	212	101	-0.32
FTA_0306	273123	273677	+	224	101	-0.35
FTA_2036	1853688	1854499	+	230	101	-0.36
FTA_0594	544296	545330	-	274	101	-0.43
FTA_1149	1038433	1039125	-	176	100	-0.25
FTA_0369	328002	328928	+	181	100	-0.26

FTA_1672	1511169	1512032	+	199	100	-0.30
FTA_1507	1348779	1349537	-	212	100	-0.33
FTA_0217	201335	202186	+	236	100	-0.37
FTA_1192	1074399	1075619	-	289	100	-0.46
trpA	93036	93785	-	180	99	-0.26
FTA_1354	1220360	1221082	+	182	99	-0.26
FTA_1762	1591743	1592447	-	205	99	-0.32
FTA_1177	1063220	1063990	+	214	99	-0.33
FTA_1228	1108413	1108976	-	218	99	-0.34
FTA_0385	337505	338341	-	219	99	-0.34
FTA_1446	1298093	1298874	+	230	99	-0.37
FTA_0039	33240	33860	-	251	99	-0.40
FTA_0411	356790	357617	+	260	99	-0.42
FTA_1783	1614247	1615374	+	316	99	-0.50
FTA_1602	1443410	1444182	-	212	98	-0.34
FTA_0654	612573	613613	+	231	98	-0.37
FTA_1959	1780462	1781088	+	247	98	-0.40
trpB	93845	94975	-	301	98	-0.49
FTA_0880	813810	816572	+	495	98	-0.70
FTA_1641	1482612	1482977	+	138	97	-0.15
udk	1459311	1459916	-	180	97	-0.27
gcvT	456838	457854	+	211	97	-0.34
FTA_0981	904750	905880	+	239	97	-0.39
FTA_1436	1290844	1291923	-	244	97	-0.40
FTA_1268	1149658	1150815	+	273	97	-0.45
purA	1856459	1857685	+	301	97	-0.49
FTA_0467	411970	412512	+	167	96	-0.24
FTA_1293	1172656	1173201	+	181	96	-0.28
FTA_1247	1131741	1132424	+	206	96	-0.33
FTA_1028	946512	947435	+	216	96	-0.35
FTA_2031	1850697	1851452	-	237	96	-0.39
FTA_1946	1769365	1770186	+	245	96	-0.41
FTA_1061	973097	974064	+	250	96	-0.42
malQ	474538	475941	+	267	96	-0.44
FTA_0064	55898	56503	-	191	95	-0.30
FTA_0803	752631	753326	-	204	95	-0.33
FTA_1068	975557	976228	+	215	95	-0.35
FTA_1085	988439	989341	+	225	95	-0.37
FTA_1654	1492995	1493609	+	227	95	-0.38
FTA_1371	1235998	1236810	-	275	95	-0.46
FTA_0229	213872	214969	-	311	95	-0.52
FTA_0452	397532	398353	+	157	94	-0.22
FTA_0916	845993	846961	+	172	94	-0.26
FTA_1503	1343990	1344394	-	192	94	-0.31
speE	487049	487858	+	205	94	-0.34

proC	533624	534388	+	206	94	-0.34
tal	1055535	1056494	+	206	94	-0.34
FTA_0183	172031	172750	-	219	94	-0.37
FTA_1336	1205743	1207065	-	344	94	-0.56
FTA_1305	1181336	1182697	+	48	93	0.29
FTA_0785	734671	735333	+	150	93	-0.21
FTA_1527	1367085	1367986	-	154	93	-0.22
FTA_1879	1702100	1702918	-	156	93	-0.22
FTA_1650	1488410	1489112	-	168	93	-0.26
pdpDII	1097229	1097783	+	176	93	-0.28
FTA_1297	1174287	1174916	-	183	93	-0.29
FTA_0988	911596	912234	-	189	93	-0.31
wbtM	596468	597424	+	197	93	-0.33
FTA_1008	926670	927494	-	199	93	-0.33
FTA_0610	557034	557738	+	213	93	-0.36
FTA_1512	1353909	1354808	-	240	93	-0.41
FTA_1758	1587360	1588502	-	349	93	-0.57
FTA_1823	1646162	1646602	+	156	92	-0.23
pdpD1	101923	102477	+	170	92	-0.27
FTA_1506	1347700	1348503	-	175	92	-0.28
FTA_1911	1732516	1733385	-	177	92	-0.28
FTA_2067	1876488	1877076	+	184	92	-0.30
FTA_0066	56966	57598	+	196	92	-0.33
FTA_0307	273744	274409	-	199	92	-0.34
FTA_0173	163948	164700	-	204	92	-0.35
truB	538446	539294	+	221	92	-0.38
FTA_1324	1196737	1198002	-	307	92	-0.52
FTA_0934	863662	864444	-	158	91	-0.24
tgt	826340	827383	+	224	91	-0.39
FTA_1009	927563	928348	-	227	91	-0.40
recX	10611	11003	+	114	90	-0.10
FTA_0129	115186	115647	-	148	90	-0.22
FTA_1269	1150878	1151564	+	173	90	-0.28
FTA_0697	646213	646806	-	174	90	-0.29
FTA_1530	1370119	1370772	+	187	90	-0.32
FTA_0690	639861	640886	+	200	90	-0.35
FTA_0598	547480	548388	-	222	90	-0.39
FTA_0692	640943	642040	+	222	90	-0.39
FTA_1546	1384069	1385037	+	242	90	-0.43
FTA_0175	165461	166018	-	121	89	-0.13
FTA_0924	852870	853310	+	136	89	-0.18
deoC	1589770	1590471	-	166	89	-0.27
galT	1326324	1327289	+	202	89	-0.36
FTA_1714	1551609	1552193	+	210	89	-0.37
FTA_0562	514189	514845	-	217	89	-0.39

FTA_0983	906629	907984	+	247	89	-0.44
thrC	484681	485913	+	304	89	-0.53
FTA_1444	1296266	1296889	+	150	88	-0.23
FTA_2004	1822651	1823109	-	162	88	-0.27
FTA_1141	1033776	1034251	-	169	88	-0.28
FTA_2030	1849237	1849959	+	191	88	-0.34
FTA_0451	396822	397469	+	202	88	-0.36
FTA_1001	921847	923046	+	246	88	-0.45
FTA_0219	202709	203650	+	251	88	-0.46
FTA_0041	35426	36406	-	268	88	-0.48
FTA_1835	1658728	1659552	+	300	88	-0.53
FTA_2069	1877380	1877799	-	126	87	-0.16
FTA_1390	1252727	1253101	-	145	87	-0.22
FTA_0131	117444	117878	-	155	87	-0.25
FTA_1743	1572978	1573529	-	172	87	-0.30
FTA_1700	1536023	1536769	+	197	87	-0.35
FTA_1596	1439246	1439989	+	206	87	-0.37
mutM	1468860	1469624	+	207	87	-0.38
FTA_1686	1525606	1526466	-	287	87	-0.52
ksgA	1518073	1518801	-	140	86	-0.21
FTA_0095	81123	81782	+	172	86	-0.30
FTA_0401	349580	350173	-	207	86	-0.38
FTA_1858	1684435	1685088	-	208	86	-0.38
FTA_0093	78917	79753	-	210	86	-0.39
FTA_0894	827664	828209	-	127	85	-0.17
FTA_1230	1110492	1110953	-	136	85	-0.20
FTA_1363	1230348	1230782	-	157	85	-0.27
FTA_0288	257453	257983	+	181	85	-0.33
FTA_0671	624772	625398	+	201	85	-0.37
msrA	1883006	1883635	-	215	85	-0.40
FTA_0807	754713	755858	+	257	85	-0.48
FTA_1064	974246	974647	+	110	84	-0.12
FTA_1571	1408062	1408613	-	167	84	-0.30
FTA_0113	99565	99993	+	189	84	-0.35
FTA_0334	302322	302909	+	196	84	-0.37
FTA_0388	339600	340230	-	230	84	-0.44
FTA_0154	148126	148473	+	135	83	-0.21
FTA_0729	678606	679373	+	155	83	-0.27
FTA_0564	514980	515426	-	159	83	-0.28
FTA_0432	377777	378370	-	171	83	-0.31
FTA_1443	1294758	1295684	-	179	83	-0.33
FTA_1591	1434302	1435023	-	220	83	-0.42
FTA_0187	174267	175079	-	237	83	-0.46
FTA_0750	702236	703129	-	244	83	-0.47
FTA_1126	1020946	1022718	+	253	83	-0.48

FTA_1479	1321310	1322959	-	318	83	-0.58
FTA_2003	1821104	1822427	-	345	83	-0.62
FTA_0615	562282	562710	-	137	82	-0.22
FTA_0888	822305	823297	-	179	82	-0.34
FTA_0793	744514	745380	+	211	82	-0.41
FTA_0910	840368	841180	-	222	82	-0.43
FTA_1941	1763377	1764579	+	227	82	-0.44
FTA_1859	1685152	1685856	-	245	82	-0.48
galK	1327352	1328440	+	269	82	-0.52
FTA_1438	1292074	1292583	-	139	81	-0.23
FTA_0221	204701	205321	+	144	81	-0.25
leuD	1816618	1817127	-	145	81	-0.25
deoD	1385547	1386209	-	173	81	-0.33
mraW	1465745	1466602	-	175	81	-0.33
FTA_0186	173786	174196	+	181	81	-0.35
FTA_1102	1000394	1001146	-	183	81	-0.35
FTA_0405	351486	352217	+	186	81	-0.36
FTA_1417	1275785	1276432	-	196	81	-0.38
FTA_1259	1142634	1143362	-	222	81	-0.44
FTA_1912	1733438	1734445	-	250	81	-0.49
FTA_1788	1618133	1619002	-	284	81	-0.54
FTA_0979	903310	903996	+	141	80	-0.25
FTA_1961	1782311	1782865	-	151	80	-0.28
FTA_1523	1365244	1365881	-	176	80	-0.34
FTA_1052	967219	967965	+	179	80	-0.35
FTA_0989	912385	912906	-	182	80	-0.36
FTA_0810	758791	759486	-	203	80	-0.40
FTA_0510	467698	469272	+	214	80	-0.43
FTA_1725	1558239	1558904	-	216	80	-0.43
FTA_1389	1251773	1252645	-	219	80	-0.44
FTA_1226	1106501	1107214	-	220	80	-0.44
FTA_0341	307508	308589	-	314	80	-0.59
FTA_0984	908223	908612	+	131	79	-0.22
FTA_0304	271748	272320	-	148	79	-0.27
FTA_1651	1490014	1490676	+	160	79	-0.31
FTA_0673	625460	626149	+	211	79	-0.43
FTA_0091	78342	78797	-	216	79	-0.44
FTA_1224	1101882	1102397	-	229	79	-0.46
FTA_1683	1520272	1522818	-	245	79	-0.49
FTA_0338	304199	305452	-	307	79	-0.59
FTA_0150	143983	145773	+	362	79	-0.66
FTA_1767	1599346	1599777	+	132	78	-0.23
nagA	774128	775201	+	173	78	-0.35
FTA_0033	25227	25970	+	176	78	-0.35
FTA_1146	1035408	1035965	+	197	78	-0.40

FTA_1409	1268321	1269634	-	202	78	-0.41
FTA_1309	1184097	1184876	+	215	78	-0.44
FTA_0046	40430	40777	-	114	77	-0.17
FTA_1090	991178	991597	+	128	77	-0.22
FTA_0544	499255	499728	-	137	77	-0.25
FTA_1818	1643401	1643775	+	166	77	-0.33
FTA_0737	687801	688538	-	173	77	-0.35
FTA_0734	685115	685780	-	199	77	-0.41
FTA_1103	1001220	1001963	-	215	77	-0.45
FTA_2072	1880710	1881078	+	126	76	-0.22
cyoC	195163	195705	+	146	76	-0.28
FTA_0897	829318	829830	+	151	76	-0.30
FTA_0605	551397	551945	+	153	76	-0.30
FTA_0660	618379	618945	-	156	76	-0.31
FTA_1327	1198823	1199456	+	166	76	-0.34
FTA_1010	928448	929206	-	188	76	-0.39
FTA_1869	1691657	1693024	+	285	76	-0.57
FTA_0446	390731	391138	+	115	75	-0.19
FTA_0104	91902	92479	+	209	75	-0.45
FTA_0125	111195	111908	-	211	75	-0.45
FTA_0855	799175	800093	+	234	75	-0.49
FTA_0908	838726	839352	+	117	74	-0.20
FTA_2032	1851582	1851932	-	154	74	-0.32
FTA_0991	913597	914169	+	168	74	-0.36
FTA_0662	619774	620430	+	172	74	-0.37
FTA_2029	1850013	1850522	-	182	74	-0.39
FTA_1240	1128113	1128942	+	188	74	-0.40
FTA_0123	106576	107091	-	207	74	-0.45
FTA_1972	1791563	1793032	-	233	74	-0.50
FTA_0148	141543	142115	-	130	73	-0.25
FTA_0043	37670	38161	+	134	73	-0.26
FTA_1237	1124952	1125476	-	193	73	-0.42
cyoA	192195	193037	+	222	73	-0.48
rumA	1456826	1458115	-	339	73	-0.67
FTA_1789	1619468	1619728	-	101	72	-0.15
FTA_0320	287526	287918	+	131	72	-0.26
FTA_0998	919176	920048	-	161	72	-0.35
FTA_0733	683736	684362	+	171	72	-0.38
FTA_1884	1705468	1706022	-	194	72	-0.43
FTA_1418	1276504	1276842	-	101	71	-0.15
FTA_1096	995654	996055	+	133	71	-0.27
FTA_0438	383954	384412	-	156	71	-0.34
FTA_0653	611680	612387	+	186	71	-0.42
FTA_1148	1037584	1038279	-	193	71	-0.43
FTA_0612	558201	559001	+	207	71	-0.46

FTA_0195	182272	182619	+	99	70	-0.15
ung	513166	513768	+	128	70	-0.26
FTA_0213	198233	198868	-	143	70	-0.31
FTA_0667	622492	623100	+	148	70	-0.33
FTA_1560	1396033	1396557	-	158	70	-0.35
FTA_0771	722775	723428	-	165	70	-0.37
FTA_1307	1183141	1183629	+	171	70	-0.39
FTA_1429	1285238	1285852	+	176	70	-0.40
msrAB	1041879	1042673	+	213	70	-0.48
FTA_1318	1190541	1191188	+	213	70	-0.48
FTA_1745	1575094	1575837	-	224	70	-0.51
FTA_1239	1127133	1127918	-	252	70	-0.56
FTA_0723	672982	673344	-	100	69	-0.16
FTA_1676	1514121	1514741	-	104	69	-0.18
FTA_0686	635771	636220	-	129	69	-0.27
FTA_0538	495248	495844	+	141	69	-0.31
gpsA	344094	345032	+	142	69	-0.31
FTA_1662	1500997	1501600	-	145	69	-0.32
FTA_1463	1310797	1311384	-	153	69	-0.35
def2	451201	451773	+	157	69	-0.36
FTA_0591	542510	543193	-	168	69	-0.39
FTA_0398	347012	347722	-	195	69	-0.45
FTA_0063	55076	55567	-	201	69	-0.46
FTA_0496	446132	446455	+	111	68	-0.21
FTA_0495	445396	445719	-	119	68	-0.24
FTA_0051	43459	43947	+	142	68	-0.32
FTA_1352	1219784	1220278	+	147	68	-0.33
FTA_1631	1471185	1471988	-	167	68	-0.39
FTA_2062	1874448	1875000	-	196	68	-0.46
FTA_0070	61321	61956	-	200	68	-0.47
ispZ	371972	372442	+	76	67	-0.05
FTA_1947	1770262	1770609	-	107	67	-0.20
FTA_1843	1666184	1667290	-	128	67	-0.28
FTA_1395	1256478	1257029	-	171	67	-0.41
FTA_0834	776723	777856	+	197	67	-0.47
FTA_1430	1285916	1286905	+	216	67	-0.51
FTA_1880	1703048	1703365	+	102	66	-0.19
ttg2	498518	499120	-	119	66	-0.26
kdpC	1808790	1809284	-	136	66	-0.31
putP	1626036	1627442	-	138	66	-0.32
FTA_1614	1456181	1456756	-	149	66	-0.35
FTA_0469	416403	416879	-	165	66	-0.40
FTA_0579	530661	531482	-	167	66	-0.40
FTA_1819	1644446	1645180	+	178	66	-0.43
uspA	168394	169170	-	194	66	-0.47

deoC	1590494	1591678	-	248	66	-0.57
FTA_0698	647035	647343	+	82	65	-0.10
FTA_1542	1380541	1381047	-	119	65	-0.26
FTA_0688	637486	637962	-	138	65	-0.33
apaH	1517234	1518001	-	166	65	-0.41
queA	720079	721035	+	204	65	-0.50
FTA_0841	783476	784420	+	245	65	-0.58
FTA_2144	361615	361995	-	116	64	-0.26
FTA_1334	1203431	1203925	-	131	64	-0.31
FTA_0002	2643	3020	-	132	64	-0.31
FTA_0159	151380	152636	+	134	64	-0.32
FTA_0488	436679	437293	+	136	64	-0.33
FTA_0592	543255	543737	-	138	64	-0.33
nth	897378	897956	-	138	64	-0.33
FTA_1809	1637250	1637735	-	142	64	-0.35
FTA_0305	272457	272975	-	144	64	-0.35
FTA_0444	389085	389474	+	144	64	-0.35
FTA_1826	1650010	1650588	+	148	64	-0.36
purN	368251	368766	+	149	64	-0.37
tdk	868955	869488	+	154	64	-0.38
FTA_1232	1112750	1113184	-	159	64	-0.40
FTA_0770	721730	722461	+	193	64	-0.48
FTA_1513	1354877	1355578	-	196	64	-0.49
FTA_0011	7914	8429	+	108	63	-0.23
FTA_1283	1164985	1165430	-	117	63	-0.27
iglAII	1097888	1098418	+	161	63	-0.41
FTA_1881	1704569	1704985	-	195	63	-0.49
FTA_1381	1244142	1245143	-	200	63	-0.50
FTA_1006	924992	926023	-	205	63	-0.51
FTA_2061	1873461	1874381	+	205	63	-0.51
FTA_0507	461357	462142	-	209	63	-0.52
FTA_1525	1365938	1366696	-	228	63	-0.56
FTA_0132	117951	118268	-	117	62	-0.28
FTA_0878	812943	813626	-	122	62	-0.29
FTA_1883	1705128	1705430	-	127	62	-0.31
FTA_1135	1030443	1031108	+	143	62	-0.36
FTA_1872	1696551	1696925	+	149	62	-0.38
FTA_1178	1064181	1065119	-	167	62	-0.43
FTA_1570	1407359	1407949	+	180	62	-0.46
FTA_0299	265742	266608	+	238	62	-0.58
FTA_0149	142826	143734	-	242	62	-0.59
FTA_1715	1552269	1552400	-	73	61	-0.08
FTA_0478	427997	428917	+	102	61	-0.22
FTA_1583	1424575	1424895	-	104	61	-0.23
FTA_0490	440384	440923	-	140	61	-0.36

FTA_0672	626202	626750	-	145	61	-0.38
FTA_1522	1364643	1365173	-	154	61	-0.40
FTA_1422	1277993	1278582	-	177	61	-0.46
FTA_0069	59574	60182	+	206	61	-0.53
FTA_0990	913094	913537	+	102	60	-0.23
FTA_0676	628059	628496	-	113	60	-0.27
rdgB	532939	533457	-	127	60	-0.33
hflC	882857	883723	+	131	60	-0.34
FTA_1050	965985	966524	+	140	60	-0.37
msrB	350252	350704	-	143	60	-0.38
xthA	1189732	1190460	+	159	60	-0.42
FTA_1143	1034487	1034981	-	161	60	-0.43
FTA_0174	164844	165401	-	164	60	-0.44
FTA_0912	841505	842062	-	165	60	-0.44
FTA_0802	751753	752304	-	169	60	-0.45
FTA_1074	980192	980383	-	82	59	-0.14
FTA_0604	550821	551162	+	122	59	-0.32
FTA_1330	1200296	1200802	+	144	59	-0.39
FTA_0833	775907	776653	+	163	59	-0.44
FTA_0658	616347	617231	-	204	59	-0.54
FTA_1368	1233567	1234331	+	224	59	-0.58
FTA_1170	1056580	1056978	+	102	58	-0.25
rrmJ	399060	399620	-	103	58	-0.25
FTA_0392	343121	343714	+	207	58	-0.55
FTA_1784	1615448	1615879	+	113	57	-0.30
FTA_0162	156286	156765	+	115	57	-0.30
FTA_0314	282196	282639	+	128	57	-0.35
FTA_1691	1529448	1530404	-	137	57	-0.38
iglA1	102618	103112	+	154	57	-0.43
FTA_1616	1458264	1458776	-	165	57	-0.46
FTA_1771	1605993	1606514	+	170	57	-0.47
FTA_0782	732810	733655	+	181	57	-0.50
FTA_1098	997142	997447	+	81	56	-0.16
FTA_0110	98165	98389	-	104	56	-0.27
FTA_0232	215842	216159	+	107	56	-0.28
FTA_0681	631460	631882	-	111	56	-0.30
FTA_0449	395260	395841	+	123	56	-0.34
FTA_1049	965308	965847	-	124	56	-0.35
FTA_1306	1182762	1183088	+	140	56	-0.40
FTA_0522	484018	484608	+	147	56	-0.42
FTA_1017	935674	936363	+	151	56	-0.43
hflK	881788	882795	+	168	56	-0.48
FTA_0987	909646	910398	+	180	56	-0.51
FTA_0383	336882	337397	-	97	55	-0.25
FTA_1125	1020340	1020873	+	97	55	-0.25

FTA_1340	1209764	1210204	+	116	55	-0.32
FTA_1104	1002016	1002372	-	118	55	-0.33
clpX	872182	873375	+	130	55	-0.37
FTA_0462	408410	409795	+	177	55	-0.51
FTA_0852	795222	796670	+	227	55	-0.62
FTA_1613	1455304	1455801	-	148	54	-0.44
FTA_0623	573609	574436	+	188	54	-0.54
FTA_0648	606966	607808	+	213	54	-0.60
FTA_1383	1245674	1246795	-	29	53	0.26
FTA_0433	378408	379175	+	55	53	-0.02
dcd	1158005	1158511	+	110	53	-0.32
FTA_0318	286546	286881	+	117	53	-0.34
FTA_0190	177454	177852	+	127	53	-0.38
FTA_1410	1269750	1270358	-	147	53	-0.44
FTA_1834	1659609	1660133	-	163	53	-0.49
FTA_0735	685850	686551	-	171	53	-0.51
FTA_0474	424878	425474	-	197	53	-0.57
FTA_0231	215019	215642	-	210	53	-0.60
FTA_0738	688591	689529	-	212	53	-0.60
FTA_0315	282680	283633	+	255	53	-0.68
FTA_1737	1568924	1569103	-	85	52	-0.21
FTA_1375	1238404	1238739	-	99	52	-0.28
FTA_1233	1113257	1113574	-	115	52	-0.34
FTA_0145	139730	140170	-	128	52	-0.39
FTA_1280	1159829	1160344	-	135	52	-0.41
FTA_1973	1793101	1793661	-	138	52	-0.42
FTA_0048	41833	42144	-	140	52	-0.43
FTA_1199	1079026	1079652	+	140	52	-0.43
FTA_1636	1476595	1477302	-	194	52	-0.57
FTA_0819	765988	766698	+	201	52	-0.59
FTA_1286	1166613	1167049	+	67	51	-0.12
trx2	1173957	1174223	-	70	51	-0.14
FTA_1100	998878	999249	+	84	51	-0.22
FTA_0100	84913	85269	+	94	51	-0.27
cdd	1409613	1409954	+	103	51	-0.31
FTA_0593	543794	544243	-	106	51	-0.32
FTA_1174	1059283	1059741	-	112	51	-0.34
FTA_0396	345093	345569	-	117	51	-0.36
FTA_0577	528970	529719	-	123	51	-0.38
FTA_1801	1628815	1629393	-	161	51	-0.50
FTA_0137	129637	130292	+	171	51	-0.53
FTA_0202	186124	186804	-	171	51	-0.53
FTA_0725	674386	675798	-	179	51	-0.55
FTA_0781	731741	732763	+	195	51	-0.58
FTA_0590	541667	542299	-	205	51	-0.60

FTA_0601	548648	548959	-	99	50	-0.30
FTA_1773	1606934	1607170	-	100	50	-0.30
FTA_0905	836593	837123	+	104	50	-0.32
FTA_0359	320256	320546	+	105	50	-0.32
FTA_0936	864975	865898	-	124	50	-0.39
FTA_0761	713996	714583	-	130	50	-0.41
upp	1444327	1444911	-	141	50	-0.45
miaB	865962	867230	-	150	50	-0.48
FTA_2073	1882588	1882707	-	51	49	-0.02
FTA_1295	1173265	1173498	+	80	49	-0.21
FTA_1985	1805289	1805600	-	92	49	-0.27
FTA_1496	1337751	1337987	-	96	49	-0.29
FTA_1005	924702	924917	-	105	49	-0.33
FTA_1790	1619901	1620314	-	110	49	-0.35
FTA_0483	433353	433814	+	127	49	-0.41
FTA_0292	261093	261605	-	148	49	-0.48
FTA_1344	1211426	1212697	-	168	49	-0.54
FTA_1445	1296973	1297632	-	198	49	-0.61
recA	9545	10564	+	234	49	-0.68
wzx	592672	594099	+	57	48	-0.07
FTA_1207	1085951	1086394	-	99	48	-0.31
FTA_1857	1683960	1684397	-	123	48	-0.41
FTA_0151	146170	146778	+	138	48	-0.46
FTA_2049	1862902	1863306	-	144	48	-0.48
FTA_1298	1175070	1175791	-	160	48	-0.52
FTA_1138	1033258	1033386	-	76	47	-0.21
FTA_0391	342613	342918	-	91	47	-0.29
FTA_1685	1524801	1525127	-	115	47	-0.39
glk	372816	373772	-	121	47	-0.41
FTA_1051	966649	967092	+	142	47	-0.48
FTA_1182	1067950	1068441	+	153	47	-0.51
kbl	1447946	1449100	+	171	47	-0.56
FTA_0485	434518	434787	-	85	46	-0.27
FTA_0871	807381	807713	+	95	46	-0.31
FTA_1675	1513751	1514035	-	103	46	-0.35
FTA_0993	915840	916241	-	110	46	-0.38
FTA_1361	1228828	1229322	-	148	46	-0.51
FTA_0746	697582	698853	+	197	46	-0.63
udhA	929270	930625	-	203	46	-0.64
cdsA	228637	229371	+	40	45	0.05
FTA_0230	213594	213815	+	73	45	-0.21
FTA_0956	883784	884209	+	78	45	-0.24
FTA_1260	1143515	1143703	+	86	45	-0.28
FTA_1195	1075679	1076407	-	115	45	-0.41
FTA_0842	784482	784985	+	120	45	-0.43

FTA_0366	326063	326518	+	129	45	-0.46
FTA_1958	1779726	1780064	+	129	45	-0.46
FTA_1709	1545362	1545790	-	145	45	-0.51
FTA_0938	867552	868127	+	157	45	-0.54
FTA_1433	1287574	1288431	-	187	45	-0.62
FTA_1349	1216351	1217106	-	206	45	-0.66
pdxY	198928	199719	-	228	45	-0.70
FTA_0296	264428	264697	+	81	44	-0.27
FTA_0165	158424	158684	+	96	44	-0.34
FTA_1832	1656457	1656864	-	109	44	-0.39
FTA_1099	998174	998737	+	113	44	-0.41
FTA_1035	953877	954419	-	124	44	-0.45
FTA_1193	1073930	1074361	+	125	44	-0.45
FTA_1087	989394	990149	+	127	44	-0.46
gidB	58960	59517	+	135	44	-0.49
FTA_0726	675868	676830	-	172	44	-0.59
FTA_0295	263832	264371	+	198	44	-0.65
FTA_0301	268963	269260	+	41	43	0.02
FTA_1679	1516793	1517170	-	73	43	-0.23
FTA_1698	1534800	1534898	+	79	43	-0.26
mglB	1134310	1134657	-	96	43	-0.35
FTA_1041	960231	960686	+	104	43	-0.38
FTA_1043	961406	961915	-	104	43	-0.38
gloA	723604	723927	+	111	43	-0.41
FTA_0372	330376	330741	-	118	43	-0.44
FTA_2152	1875166	1875519	+	118	43	-0.44
FTA_0784	733726	734156	-	129	43	-0.48
FTA_0218	202236	202652	+	143	43	-0.52
FTA_1509	1351188	1351790	-	186	43	-0.64
FTA_0753	704886	706022	-	46	42	-0.04
FTA_0282	251302	251391	+	63	42	-0.18
FTA_0331	301183	301260	-	69	42	-0.22
gcvH1	457971	458294	+	73	42	-0.24
FTA_1191	1073568	1073777	+	77	42	-0.26
FTA_0524	486561	486989	+	88	42	-0.32
FTA_1322	1193883	1194143	+	98	42	-0.37
minD	501648	502412	-	131	42	-0.49
FTA_0760	713222	713860	+	148	42	-0.55
FTA_1514	1355642	1356277	-	161	42	-0.58
FTA_1955	1776630	1777148	-	165	42	-0.59
sucA	1710760	1713525	-	15	41	0.44
pyrC	34052	35338	+	60	41	-0.17
FTA_1053	968131	968337	+	71	41	-0.24
pyrF	47715	48287	+	75	41	-0.26
FTA_0674	626862	627251	-	99	41	-0.38

FTA_1471	1314677	1315063	+	99	41	-0.38
FTA_1469	1313734	1314144	-	105	41	-0.41
FTA_2077	1884550	1884930	-	105	41	-0.41
FTA_0023	16932	17168	-	108	41	-0.42
rph	333604	334251	+	146	41	-0.55
FTA_1133	1029096	1029665	+	146	41	-0.55
FTA_1069	976291	977100	-	176	41	-0.63
FTA_1310	1184933	1185139	+	71	40	-0.25
FTA_1780	1611530	1611721	+	73	40	-0.26
FTA_0621	570983	571270	+	88	40	-0.34
FTA_1097	996540	997030	+	89	40	-0.35
FTA_1637	1478030	1478284	+	97	40	-0.38
cyoD	195767	196039	+	104	40	-0.41
FTA_0575	526775	527089	+	118	40	-0.47
FTA_0879	812202	812894	+	121	40	-0.48
FTA_0731	682937	683605	-	155	40	-0.59
recR	1339984	1340526	+	155	40	-0.59
FTA_1608	1450240	1451442	+	73	39	-0.27
FTA_1431	1286987	1287220	+	87	39	-0.35
FTA_0843	785039	785575	+	100	39	-0.41
FTA_0424	371546	371896	-	127	39	-0.51
FTA_0923	852225	852344	+	43	38	-0.05
FTA_0198	183862	183996	+	60	38	-0.20
FTA_1470	1314233	1314520	-	77	38	-0.31
FTA_1151	1041274	1041690	-	78	38	-0.31
FTA_0675	627542	627982	-	90	38	-0.37
FTA_1569	1406747	1407265	+	91	38	-0.38
FTA_0054	48337	49035	+	92	38	-0.38
fba	1092291	1093295	+	101	38	-0.42
FTA_0679	629984	630337	+	109	38	-0.46
FTA_1367	1232960	1233370	+	110	38	-0.46
FTA_0517	478679	479014	+	118	38	-0.49
FTA_0702	653369	653839	+	141	38	-0.57
FTA_1630	1470643	1471131	-	149	38	-0.59
aroE	176554	177267	-	189	38	-0.70
FTA_1772	1606711	1606779	-	34	37	0.04
FTA_1160	1046802	1047149	-	73	37	-0.30
FTA_0169	159896	160183	-	79	37	-0.33
FTA_0744	695540	696028	+	100	37	-0.43
FTA_0973	898640	899224	-	107	37	-0.46
FTA_1579	1418871	1419320	-	119	37	-0.51
FTA_1339	1208828	1209688	+	127	37	-0.54
sodC	350925	351422	+	156	37	-0.62
FTA_1137	1032389	1033022	-	165	37	-0.65
pckA	1540474	1542006	-	191	37	-0.71

FTA_0749	701095	702099	+	27	36	0.12
FTA_1147	1036853	1037146	-	68	36	-0.28
FTA_0552	503785	504069	-	77	36	-0.33
FTA_2075	1883779	1884087	-	79	36	-0.34
FTA_2076	1884140	1884421	-	83	36	-0.36
wbtI	802460	591885	+	89	36	-0.39
FTA_0722	672428	672823	+	89	36	-0.39
FTA_0713	666768	667085	+	112	36	-0.49
FTA_0416	362965	363624	+	153	36	-0.63
FTA_1669	1507551	1508597	+	40	35	-0.06
truA	1022781	1023497	+	61	35	-0.24
FTA_1315	1188892	1189065	-	77	35	-0.34
panB	662915	663652	-	85	35	-0.39
FTA_1161	1047240	1047515	+	86	35	-0.39
FTA_1316	1189116	1189388	-	88	35	-0.40
FTA_1534	1373075	1373389	-	94	35	-0.43
FTA_0157	148886	149203	+	100	35	-0.46
ruvC	904153	904608	+	106	35	-0.48
FTA_1490	1331619	1332263	-	108	35	-0.49
FTA_0445	390195	390638	+	122	35	-0.54
tdh	1449183	1450178	+	151	35	-0.63
FTA_1667	1504775	1505896	-	240	35	-0.84
FTA_2150	1072491	1072550	+	28	34	0.08
folK	1207550	1208755	+	38	34	-0.05
FTA_1432	1287365	1287511	+	62	34	-0.26
metG	413809	415773	-	63	34	-0.27
nusB	628636	629001	+	76	34	-0.35
FTA_0762	714699	715265	-	80	34	-0.37
cmk	979065	980057	-	90	34	-0.42
secB2	278008	278391	-	94	34	-0.44
bioD	1215584	1216183	+	118	34	-0.54
FTA_0308	274469	275287	-	149	34	-0.64
FTA_0626	580107	581783	+	199	34	-0.77
FTA_0356	317952	318143	+	41	33	-0.09
FTA_1054	968470	968625	+	44	33	-0.12
rpsU	1293511	1293651	-	59	33	-0.25
FTA_1180	1066226	1066423	+	64	33	-0.29
FTA_2060	1873203	1873355	-	69	33	-0.32
rnhA	837158	837556	+	72	33	-0.34
FTA_0291	260658	260837	-	75	33	-0.36
FTA_0105	92628	92921	+	76	33	-0.36
FTA_1794	1622555	1622842	-	76	33	-0.36
crcB	147738	148013	+	79	33	-0.38
FTA_1601	1442975	1443259	-	84	33	-0.41
FTA_0603	550167	550643	+	98	33	-0.47

FTA_1945	1768754	1769056	+	116	33	-0.55
FTA_0215	199786	200160	-	122	33	-0.57
FTA_0927	855441	856088	+	136	33	-0.62
FTA_1434	1288503	1289315	-	199	33	-0.78
FTA_2151	1494784	1494861	+	29	32	0.04
FTA_1526	1366744	1367013	-	55	32	-0.24
pyrE	493095	493745	-	87	32	-0.43
FTA_0976	900916	901356	-	87	32	-0.43
FTA_0978	902635	903234	+	92	32	-0.46
FTA_0844	785641	786171	+	100	32	-0.49
FTA_1860	1685926	1687398	-	236	32	-0.87
FTA_0453	398410	398625	+	54	31	-0.24
FTA_1031	950727	950957	+	62	31	-0.30
FTA_0335	302987	303280	+	70	31	-0.35
FTA_1030	950294	950620	+	83	31	-0.43
minC	502512	503138	-	116	31	-0.57
hpt	1857749	1858222	+	127	31	-0.61
FTA_0555	507053	508507	+	43	30	-0.16
acnA	1699182	1701935	-	44	30	-0.17
FTA_1939	1760775	1761137	+	60	30	-0.30
FTA_0347	311592	311996	+	64	30	-0.33
FTA_0863	802490	802645	-	70	30	-0.37
FTA_1425	1280927	1281103	-	76	30	-0.40
FTA_1531	1370822	1371031	+	79	30	-0.42
ubiC	332999	333445	-	90	30	-0.48
FTA_1374	1237946	1238335	-	105	30	-0.54
FTA_1687	1526787	1527194	-	116	30	-0.59
hslU	933556	934863	-	135	30	-0.65
FTA_2146	635201	635329	-	49	29	-0.23
FTA_0721	672307	672390	+	49	29	-0.23
FTA_0168	159704	159829	+	51	29	-0.25
FTA_0009	6660	6803	-	57	29	-0.29
FTA_1329	1199656	1200027	+	66	29	-0.36
FTA_1214	1094978	1095175	-	73	29	-0.40
FTA_0768	719592	719993	+	79	29	-0.44
FTA_0112	98883	99215	-	80	29	-0.44
FTA_0823	768549	768920	+	80	29	-0.44
FTA_1071	977785	978336	+	102	29	-0.55
FTA_0857	800262	800432	-	51	28	-0.26
secF	832814	833698	+	52	28	-0.27
FTA_1439	1293226	1293393	+	55	28	-0.29
FTA_0779	729311	729643	-	58	28	-0.32
FTA_1065	974754	975127	+	74	28	-0.42
FTA_0862	801866	802231	-	82	28	-0.47
wzy	586658	587827	+	178	28	-0.80

wbtK	594152	594952	+	26	27	0.02
wbtD	583251	584282	+	45	27	-0.22
FTA_0806	754366	754611	+	49	27	-0.26
FTA_0710	663714	664313	-	51	27	-0.28
FTA_1067	975271	975483	+	55	27	-0.31
aroH	312995	313297	+	63	27	-0.37
FTA_1724	1557924	1558112	-	74	27	-0.44
FTA_1185	1069913	1070239	+	84	27	-0.49
FTA_0650	609528	609953	-	88	27	-0.51
FTA_1484	1328497	1328832	-	88	27	-0.51
FTA_1403	1264015	1265136	-	176	27	-0.81
FTA_0578	529849	530553	-	187	27	-0.84
FTA_2138	1644263	1644302	-	5	26	0.72
FTA_0663	620683	620775	+	36	26	-0.14
FTA_0441	386206	386394	-	65	26	-0.40
FTA_1089	990612	990992	+	88	26	-0.53
dtd	50558	50935	-	97	26	-0.57
FTA_0111	98452	98802	-	103	26	-0.60
FTA_2052	1865186	1865701	+	115	26	-0.65
FTA_0097	82602	83294	-	28	25	-0.05
FTA_1421	1276982	1277944	+	30	25	-0.08
rpoC	1668813	1673006	-	32	25	-0.11
FTA_0032	24511	24684	-	69	25	-0.44
FTA_1164	1049484	1049870	-	83	25	-0.52
FTA_0985	909026	909307	-	95	25	-0.58
FTA_0970	896950	897321	-	103	25	-0.61
FTA_1395	1257037	1257270	-	104	25	-0.62
wbtC	582470	583201	+	130	25	-0.72
xerD	1661166	1661984	-	132	25	-0.72
wbtE	584360	585610	+	9	24	0.43
FTA_1508	1349800	1350846	+	15	24	0.20
coaBC	798003	799118	+	35	24	-0.16
FTA_1461	1310519	1310638	-	41	24	-0.23
FTA_1736	1568629	1568850	+	42	24	-0.24
FTA_0868	805895	805984	-	51	24	-0.33
FTA_1139	1033558	1033701	-	52	24	-0.34
FTA_2059	1872991	1873131	-	52	24	-0.34
FTA_0892	825813	825974	-	58	24	-0.38
tig	870211	871467	+	95	24	-0.60
FTA_0497	446872	447333	+	110	24	-0.66
FTA_2136	1124518	1124572	+	25	23	-0.04
FTA_0379	334365	334628	-	29	23	-0.10
wbtH	588984	590810	+	37	23	-0.21
rpsU	433871	434008	-	38	23	-0.22
FTA_2141	22240	22350	-	41	23	-0.25

FTA_0319	287236	287304	-	41	23	-0.25
FTA_0554	506356	506439	+	47	23	-0.31
FTA_0836	779090	779293	+	54	23	-0.37
FTA_0871	807161	807361	+	63	23	-0.44
FTA_0883	818840	819538	-	69	23	-0.48
FTA_1776	1609261	1609554	-	73	23	-0.50
FTA_0922	851926	852105	+	76	23	-0.52
panC	662143	662868	-	89	23	-0.59
FTA_1640	1481630	1482442	+	93	23	-0.61
FTA_0972	898009	898578	-	96	23	-0.62
FTA_1058	970928	971341	+	107	23	-0.67
apt	1708698	1709165	-	112	23	-0.69
FTA_2148	723987	724319	+	113	23	-0.69
FTA_0461	406723	408333	+	146	23	-0.80
galU	1289964	1290767	-	153	23	-0.82
gatB	1770684	1772045	-	26	22	-0.07
FTA_0798	749940	750080	+	29	22	-0.12
def1	71526	71990	-	32	22	-0.16
FTA_1032	951069	951323	+	38	22	-0.24
FTA_0185	173521	173637	+	52	22	-0.37
FTA_0965	889908	890165	+	52	22	-0.37
trpR	1888197	1888424	-	62	22	-0.45
FTA_2018	1838704	1839105	-	65	22	-0.47
wbtJ	591942	592607	+	67	22	-0.48
FTA_2087	1706157	1706182	-	15	21	0.15
FTA_0225	208195	208572	+	26	21	-0.09
rbfA	1737086	1737457	-	28	21	-0.12
topA	392767	395001	-	38	21	-0.26
FTA_0614	560632	560799	+	54	21	-0.41
FTA_1537	1377794	1378111	-	62	21	-0.47
sucC	1480435	1481538	+	110	21	-0.72
FTA_0715	668227	668973	-	165	21	-0.90
sufE	638729	639085	+	22	20	-0.04
FTA_2134	129212	129266	+	25	20	-0.10
wbtL	595037	595861	+	31	20	-0.19
FTA_2143	323078	323233	+	40	20	-0.30
FTA_0556	508571	508651	+	41	20	-0.31
FTA_1092	992828	992974	+	47	20	-0.37
FTA_1288	1167286	1167597	-	47	20	-0.37
FTA_0542	498225	498458	-	57	20	-0.45
FTA_0479	428986	429969	+	64	20	-0.51
gmhA	65979	66515	+	71	20	-0.55
FTA_0666	622103	622366	+	81	20	-0.61
greA	1395517	1395939	-	82	20	-0.61
FTA_0754	706083	707258	-	142	20	-0.85

FTA_1204	1083512	1084423	-	6	19	0.50
FTA_1918	1741586	1741984	-	21	19	-0.04
FTA_0431	377279	377350	-	25	19	-0.12
FTA_0968	894889	895515	-	29	19	-0.18
gyrA	515784	518330	+	33	19	-0.24
FTA_1822	1646653	1647447	-	36	19	-0.28
FTA_1157	1045335	1045565	-	42	19	-0.34
FTA_0184	172882	173385	-	46	19	-0.38
FTA_0559	509774	509998	+	73	19	-0.58
FTA_0864	802760	802897	-	76	19	-0.60
engB	884266	884799	+	83	19	-0.64
FTA_1733	1566005	1566688	+	92	19	-0.69
FTA_0375	331771	332070	-	100	19	-0.72
trpS	291760	292704	-	13	18	0.14
FTA_1362	1230104	1230166	+	17	18	0.02
FTA_2135	422199	422253	+	28	18	-0.19
FTA_2147	638100	638159	+	30	18	-0.22
FTA_1442	1294484	1294627	+	34	18	-0.28
FTA_0898	830009	830122	+	35	18	-0.29
FTA_0475	425787	425855	+	36	18	-0.30
FTA_0643	598762	600108	+	44	18	-0.39
FTA_1000	921461	921691	+	44	18	-0.39
FTA_0663	620796	620948	+	50	18	-0.44
nusA	1740099	1741508	-	51	18	-0.45
FTA_0393	343786	343857	-	60	18	-0.52
FTA_1404	1265303	1265488	-	64	18	-0.55
FTA_1132	1028739	1029014	+	65	18	-0.56
FTA_1198	1079702	1079956	-	80	18	-0.65
FTA_1105	1002448	1002999	-	85	18	-0.67
FTA_1459	1308594	1308902	+	89	18	-0.69
FTA_0716	669047	669868	-	185	18	-1.01
rpiA	727251	727865	-	16	17	0.03
FTA_0457	399838	400245	+	31	17	-0.26
FTA_0050	42368	42445	+	46	17	-0.43
FTA_0541	497972	498148	-	47	17	-0.44
clpP	871553	872098	+	48	17	-0.45
FTA_0454	398827	398940	+	61	17	-0.55
FTA_0932	861651	861866	-	68	17	-0.60
lepA	67555	69279	+	159	17	-0.97
FTA_1408	1266995	1268158	-	6	16	0.43
FTA_0192	178714	180309	+	23	16	-0.16
mglA	1134719	1135276	-	30	16	-0.27
purL	1784143	1787955	-	31	16	-0.29
FTA_0960	886297	886587	+	62	16	-0.59
wbtF	585658	586581	+	102	16	-0.80

FTA_2119	419047	419063	+	19	15	-0.10
gltX	217913	219259	+	24	15	-0.20
murC	175132	176427	-	33	15	-0.34
aroB	788669	789688	+	37	15	-0.39
FTA_0341	308508	308669	+	41	15	-0.44
FTA_0765	716589	717065	+	42	15	-0.45
era	778030	778863	+	44	15	-0.47
FTA_1730	1563713	1563814	+	47	15	-0.50
FTA_0861	801551	801694	-	50	15	-0.52
FTA_0484	434068	434211	-	51	15	-0.53
gcvH2	1338037	1338357	-	71	15	-0.68
rpsA	1839259	1840923	+	78	15	-0.72
ruvA	905953	906543	+	81	15	-0.73
ruvB	1031232	1032218	+	121	15	-0.91
FTA_0881	816658	818283	+	12	14	0.07
nadB	1317213	1318640	-	14	14	0.00
FTA_1808	1635781	1637175	+	15	14	-0.03
FTA_1682	1518863	1520230	-	17	14	-0.08
FTA_0820	766849	766977	+	22	14	-0.20
FTA_2050	1863377	1863913	-	22	14	-0.20
FTA_1244	1130596	1130781	-	27	14	-0.29
rpoZ	1459100	1459258	-	35	14	-0.40
FTA_1804	1631953	1634394	+	39	14	-0.44
secA	1381217	1383877	-	41	14	-0.47
FTA_1499	1339646	1339924	+	45	14	-0.51
FTA_0351	314192	314338	-	46	14	-0.52
FTA_1742	1572832	1572897	+	49	14	-0.54
FTA_0685	635523	635660	-	59	14	-0.62
FTA_1810	1637818	1638459	-	59	14	-0.62
FTA_1286	1166068	1166592	-	80	14	-0.76
FTA_0012	8625	8987	+	101	14	-0.86
FTA_0747	698939	699769	-	118	14	-0.93
FTA_1529	1369051	1369773	-	13	13	0.00
guaB	1398946	1400346	-	14	13	-0.03
rpoB	1673128	1677153	-	14	13	-0.03
minE	501373	501585	-	17	13	-0.12
hemH	806029	806967	-	17	13	-0.12
trmD	1662557	1663261	-	18	13	-0.14
cysS	1611849	1613183	+	24	13	-0.27
miaA	877690	878556	+	28	13	-0.33
FTA_1806	1635086	1635157	-	31	13	-0.38
valS	208764	211463	+	32	13	-0.39
FTA_0156	149260	149373	-	36	13	-0.44
FTA_1548	1385101	1385490	+	36	13	-0.44
FTA_1188	1072207	1072323	+	38	13	-0.47

efp	182719	183228	+	42	13	-0.51
FTA_0788	738987	739757	+	42	13	-0.51
priA	1865758	1867851	+	45	13	-0.54
FTA_1183	1068663	1068764	+	48	13	-0.57
FTA_1055	969094	969240	+	52	13	-0.60
FTA_0336	303364	303639	-	76	13	-0.77
trmE	1129238	1130530	-	104	13	-0.90
FTA_2095	1680802	1680817	-	6	12	0.30
tkt	1086593	1088524	+	12	12	0.00
FTA_2120	1121366	1121382	+	18	12	-0.18
glnA	1824352	1825329	+	18	12	-0.18
purCD	365935	368187	+	25	12	-0.32
FTA_1038	956872	957072	-	27	12	-0.35
FTA_1897	1719821	1719895	-	27	12	-0.35
FTA_1359	1226100	1226417	-	31	12	-0.41
FTA_0158	149562	151301	+	32	12	-0.43
FTA_1108	1004977	1005363	+	32	12	-0.43
FTA_1458	1308449	1308517	+	32	12	-0.43
FTA_0946	875871	876083	+	34	12	-0.45
FTA_1406	1266053	1266544	-	41	12	-0.53
FTA_0918	848837	849085	+	52	12	-0.64
FTA_1452	1301676	1302431	-	54	12	-0.65
FTA_0925	853882	854064	-	64	12	-0.73
FTA_1267	1149085	1149456	-	67	12	-0.75
FTA_0430	376924	377202	+	73	12	-0.78
thrS	1333540	1335384	-	8	11	0.14
FTA_1980	1799835	1801649	-	11	11	0.00
FTA_0500	452496	453740	+	13	11	-0.07
FTA_2137	451897	451951	+	15	11	-0.13
accC	1514895	1516190	-	15	11	-0.13
FTA_0935	864494	864922	-	16	11	-0.16
FTA_0016	11236	11652	+	17	11	-0.19
FTA_1971	1790913	1791497	-	17	11	-0.19
FTA_0694	642950	643597	-	19	11	-0.24
FTA_2113	1229514	1229530	+	21	11	-0.28
FTA_2118	126060	126076	+	22	11	-0.30
FTA_1171	1057250	1057342	-	24	11	-0.34
FTA_1187	1071682	1071849	-	31	11	-0.45
dnaQ	970218	970874	+	32	11	-0.46
gshA	1241020	1242465	-	32	11	-0.46
FTA_0915	844517	845731	-	33	11	-0.48
FTA_0406	353796	354098	+	38	11	-0.54
FTA_0380	334793	334984	-	50	11	-0.66
FTA_0763	715332	715538	-	55	11	-0.70
mdh	959112	960011	-	12	10	-0.08

FTA_1246	1131293	1131688	+	14	10	-0.15
FTA_0695	643850	643930	+	15	10	-0.18
FTA_1070	977258	977722	+	15	10	-0.18
aspS	17365	19077	-	17	10	-0.23
glnS	1542211	1543797	+	18	10	-0.26
smpB	753387	753800	-	19	10	-0.28
glyQ	476093	476923	+	20	10	-0.30
dnaB	985966	987303	+	20	10	-0.30
aroA	835313	836530	+	21	10	-0.32
FTA_1175	1060409	1061095	+	24	10	-0.38
FTA_1276	1157133	1157933	+	27	10	-0.43
FTA_1242	1129034	1129168	+	30	10	-0.48
FTA_0337	304027	304116	+	35	10	-0.54
FTA_0902	833790	834260	+	39	10	-0.59
FTA_1552	1388250	1390136	-	42	10	-0.62
FTA_0574	526097	526624	+	44	10	-0.64
FTA_1303	1179341	1179691	-	51	10	-0.71
FTA_1441	1294088	1294225	+	53	10	-0.72
panD	661814	662089	-	66	10	-0.82
FTA_0717	669928	670989	-	139	10	-1.14
rplR	241383	241676	+	6	9	0.18
FTA_0493	442326	444209	+	8	9	0.05
FTA_2104	292864	292878	-	9	9	0.00
FTA_0889	823344	823898	-	12	9	-0.12
FTA_0568	519559	521877	+	14	9	-0.19
FTA_0778	727927	729237	-	14	9	-0.19
hemL	1222891	1224126	-	15	9	-0.22
proS	633220	634857	-	16	9	-0.25
FTA_0999	920393	921301	+	16	9	-0.25
FTA_0324	291033	291698	-	17	9	-0.28
pal	318222	318785	+	18	9	-0.30
sucB	1709265	1710674	-	21	9	-0.37
FTA_0687	637017	637091	+	22	9	-0.39
FTA_0394	343919	343996	+	23	9	-0.41
ubiA	332127	332936	-	25	9	-0.44
purH	1854735	1856222	-	25	9	-0.44
FTA_2066	1876337	1876420	+	25	9	-0.44
dnaE	447643	451062	+	30	9	-0.52
FTA_0848	789806	789871	+	32	9	-0.55
FTA_0071	62175	62246	+	34	9	-0.58
FTA_0460	404743	406497	+	44	9	-0.69
FTA_0757	708960	711431	-	46	9	-0.71
FTA_1480	1323054	1323272	-	52	9	-0.76
FTA_0286	254658	255947	-	116	9	-1.11
FTA_0098	83517	83780	-	8	8	0.00

FTA_0767	718335	719510	+	9	8	-0.05
FTA_1621	1461201	1463222	-	11	8	-0.14
prfB	1830793	1831710	-	12	8	-0.18
nadE	673508	674197	-	13	8	-0.21
FTA_1844	1667464	1668414	-	13	8	-0.21
FTA_0193	180376	181215	+	14	8	-0.24
glmU	430043	431350	+	14	8	-0.24
FTA_0758	711741	712856	+	14	8	-0.24
kdsA	1459987	1460775	-	15	8	-0.27
FTA_0487	435746	436534	+	16	8	-0.30
FTA_1121	1017484	1017972	+	16	8	-0.30
tmk	1586718	1587287	-	16	8	-0.30
adk	782693	783289	-	18	8	-0.35
FTA_1487	1329630	1329818	-	18	8	-0.35
aroC	348381	349379	-	19	8	-0.38
FTA_0327	293804	294880	+	20	8	-0.40
FTA_0443	388633	388944	+	20	8	-0.40
trkA	1179904	1181217	+	20	8	-0.40
hisS	1735813	1737018	-	20	8	-0.40
ligA	664735	666711	+	22	8	-0.44
FTA_0830	773193	774005	-	22	8	-0.44
FTA_1811	1638539	1638628	-	23	8	-0.46
fabD	1082544	1083404	-	24	8	-0.48
FTA_1845	1668474	1668650	-	28	8	-0.54
FTA_0622	571379	573535	+	29	8	-0.56
alaS	1050086	1052623	+	29	8	-0.56
FTA_0343	308764	308835	+	46	8	-0.76
FTA_0155	148673	148774	+	47	8	-0.77
pgk	1089642	1090760	+	76	8	-0.98
gor	1192511	1193812	+	88	8	-1.04
nuoL	1749163	1751112	-	2	7	0.54
atpD	1724585	1725901	-	4	7	0.24
tolQ	314568	315215	+	6	7	0.07
aceF	297923	299458	+	8	7	-0.06
ribAB	72500	73651	-	9	7	-0.11
lpxK	1595316	1596224	-	10	7	-0.15
FTA_1101	999399	1000316	+	11	7	-0.20
lolA	1634455	1635012	+	11	7	-0.20
rpsT	67013	67225	-	12	7	-0.23
murA	382320	383657	-	12	7	-0.23
FTA_1285	1165656	1166006	-	12	7	-0.23
FTA_1623	1463711	1465345	-	12	7	-0.23
glmS	431431	433209	+	13	7	-0.27
obgE	1801716	1802660	-	13	7	-0.27
rpoH	834344	835162	+	14	7	-0.30

mtnN	439711	440337	+	16	7	-0.36
FTA_1119	1015381	1016655	-	16	7	-0.36
FTA_1894	1716472	1716885	-	16	7	-0.36
FTA_0739	689677	690441	+	17	7	-0.39
ftsW	1536837	1537982	-	20	7	-0.46
holA	1823255	1824172	+	20	7	-0.46
FTA_1581	1421535	1423124	+	22	7	-0.50
dnaN	1543	2586	+	23	7	-0.52
trx1	604082	604345	-	24	7	-0.54
rpe	1362863	1363471	-	27	7	-0.59
FTA_0718	671194	671271	+	28	7	-0.60
FTA_1004	924433	924501	+	29	7	-0.62
FTA_1211	1090836	1092212	+	33	7	-0.67
rnc	537767	538399	+	35	7	-0.70
FTA_1245	1130960	1131196	+	35	7	-0.70
secD	830858	832735	+	36	7	-0.71
FTA_1501	1340594	1342657	-	43	7	-0.79
FTA_1896	1718551	1719756	+	45	7	-0.81
mpl	493881	495191	+	52	7	-0.87
secB1	9074	9463	+	55	7	-0.90
FTA_1016	934931	935443	-	74	7	-1.02
FTA_1209	1088565	1089563	+	81	7	-1.06
rpmA	1378558	1378752	-	5	6	0.08
pgsA	229891	230427	+	9	6	-0.18
purM	364892	365875	+	9	6	-0.18
FTA_0832	775265	775843	+	10	6	-0.22
sdhB	1713603	1714244	-	10	6	-0.22
FTA_1931	1752604	1753554	-	10	6	-0.22
lpdA	299532	300884	+	11	6	-0.26
FTA_0659	617482	618312	+	12	6	-0.30
lipB	1014434	1014994	-	12	6	-0.30
sdhA	1714322	1716055	-	12	6	-0.30
aceE	295224	297845	+	13	6	-0.34
wbtG	587884	588924	+	14	6	-0.37
FTA_1909	1730702	1731073	-	15	6	-0.40
lipA	901622	902545	+	16	6	-0.43
FTA_1580	1419757	1421478	+	16	6	-0.43
lolD	453793	454428	+	17	6	-0.45
FTA_1018	936426	937268	-	20	6	-0.52
ubiB	375260	376858	+	21	6	-0.54
pth	926102	926617	-	21	6	-0.54
eno	1452484	1453794	-	21	6	-0.54
FTA_2101	1643846	1643861	+	27	6	-0.65
murB	380191	380979	-	29	6	-0.68
rpmG	503233	503328	-	30	6	-0.70

coaD	1265553	1265981	-	30	6	-0.70
FTA_1976	1796768	1798135	+	31	6	-0.71
dut	229441	229827	+	32	6	-0.73
FTA_0340	307321	307398	+	34	6	-0.75
FTA_1023	941176	941292	+	52	6	-0.94
ribE	73704	74249	-	4	5	0.10
FTA_2140	269361	269590	+	4	5	0.10
FTA_2097	419138	419153	+	6	5	-0.08
ispF	818348	818767	-	6	5	-0.08
FTA_1123	1018653	1019426	+	6	5	-0.08
pheT	1146670	1148982	+	6	5	-0.08
msbA	1596290	1598059	-	6	5	-0.08
FTA_1036	955633	956805	-	8	5	-0.20
FTA_1377	1239740	1240411	+	8	5	-0.20
FTA_1807	1635466	1635609	-	8	5	-0.20
FTA_1200	1080067	1081266	-	9	5	-0.26
FTA_0170	160490	161425	-	10	5	-0.30
FTA_2092	327510	327539	+	10	5	-0.30
FTA_0759	712909	713178	+	10	5	-0.30
FTA_0849	790097	790420	-	11	5	-0.34
pheS	1145636	1146589	+	11	5	-0.34
lpxH	492389	493045	-	12	5	-0.38
murG	1338521	1339576	+	14	5	-0.45
ispD	1451507	1452136	-	14	5	-0.45
FTA_1803	1631163	1631771	-	14	5	-0.45
ftsA	1835766	1836968	-	14	5	-0.45
rplS	1662210	1662497	-	15	5	-0.48
ispH	312037	312933	+	16	5	-0.51
secG	1706251	1706544	-	16	5	-0.51
ftsZ	1834577	1835662	-	16	5	-0.51
FTA_0850	790694	790921	+	17	5	-0.53
accD	1246870	1247718	-	17	5	-0.53
purB	1777242	1778480	-	17	5	-0.53
rho	602776	603978	-	18	5	-0.56
FTA_0415	362174	362521	-	20	5	-0.60
coaE	293061	293615	-	21	5	-0.62
ribF	403672	404532	-	22	5	-0.64
lpxB	524896	525978	+	22	5	-0.64
FTA_0853	796806	797930	+	22	5	-0.64
argS	1523004	1524689	+	22	5	-0.64
glyS	1282961	1284985	-	25	5	-0.70
FTA_2020	1841100	1841582	+	28	5	-0.75
FTA_1694	1531499	1531732	+	33	5	-0.82
FTA_0516	477194	478597	+	35	5	-0.85
FTA_2022	1842662	1843492	-	40	5	-0.90

dnaJ	1141442	1142560	+	43	5	-0.93
FTA_1734	1566745	1566921	+	48	5	-0.98
gidA	729715	731538	-	52	5	-1.02
atpC	1724135	1724512	-	53	5	-1.03
FTA_1255	1137545	1138636	-	2	4	0.30
nusG	1679686	1680159	-	3	4	0.12
FTA_1562	1398227	1398820	-	4	4	0.00
guaA	1025244	1026734	+	6	4	-0.18
glmM	1707356	1708627	-	6	4	-0.18
purE	368943	369374	+	7	4	-0.24
ileS	400772	403519	-	8	4	-0.30
FTA_0569	522073	522516	+	8	4	-0.30
FTA_0766	717134	718285	+	8	4	-0.30
rpsU	1004719	1004856	+	8	4	-0.30
rpsI	1135396	1135734	-	8	4	-0.30
purF	1788015	1789445	-	8	4	-0.30
FTA_2086	1347196	1347218	-	9	4	-0.35
FTA_2096	126151	126166	+	10	4	-0.40
prfA	1647500	1648525	-	10	4	-0.40
FTA_1382	1245286	1245636	-	11	4	-0.44
FTA_1899	1720735	1721004	+	11	4	-0.44
FTA_1925	1746081	1747478	-	12	4	-0.48
FTA_2139	22485	22842	-	13	4	-0.51
rpmE	1240554	1240751	+	16	4	-0.60
pyrG	1248309	1249889	-	16	4	-0.60
FTA_2016	1837130	1837753	-	16	4	-0.60
FTA_0518	479427	480725	+	18	4	-0.65
infB	1737520	1740000	-	18	4	-0.65
FTA_1461	1310658	1310726	+	19	4	-0.68
folA	224132	224614	-	20	4	-0.70
alr	1273062	1274099	-	20	4	-0.70
hemB	1527258	1528172	-	21	4	-0.72
metK	1664812	1665912	+	21	4	-0.72
rpoA2	607956	608849	+	22	4	-0.74
FTA_0644	600199	601623	+	23	4	-0.76
FTA_0949	878727	878996	+	24	4	-0.78
FTA_0969	895691	896890	-	24	4	-0.78
folE	1198189	1198749	+	25	4	-0.80
gyrB	1472178	1474535	-	25	4	-0.80
FTA_0322	290833	290910	+	27	4	-0.83
ffh	1185280	1186596	+	30	4	-0.88
FTA_1346	1213738	1214805	+	38	4	-0.98
FTA_1830	1655223	1655687	-	38	4	-0.98
atpG	1725971	1726807	-	1	3	0.48
FTA_1929	1751492	1752037	-	1	3	0.48

nadC	1318703	1319506	-	2	3	0.18
lpxD	522595	523548	+	3	3	0.00
nadA	1319575	1320540	-	3	3	0.00
FTA_1924	1745811	1745945	-	3	3	0.00
FTA_1930	1752102	1752530	-	3	3	0.00
aroK	788142	788612	+	4	3	-0.12
FTA_1118	1015064	1015288	-	4	3	-0.12
FTA_2017	1837810	1838640	-	4	3	-0.12
cydB	187057	188178	-	5	3	-0.22
rplB	235685	236449	+	5	3	-0.22
lepB	536897	537700	+	5	3	-0.22
FTA_2088	222989	223004	+	6	3	-0.30
nuoD	1757779	1758972	-	6	3	-0.30
ipk	157408	158175	+	7	3	-0.37
FTA_0417	363824	364612	+	7	3	-0.37
murA	381047	382291	-	7	3	-0.37
dnaK	1139488	1141356	+	7	3	-0.37
FTA_0162	156788	157360	+	8	3	-0.43
gltA	1717188	1718402	+	8	3	-0.43
tyrS	937391	938572	+	9	3	-0.48
nrdA	957136	958848	-	9	3	-0.48
FTA_1407	1266618	1266914	-	9	3	-0.48
FTA_1624	1465398	1465688	-	9	3	-0.48
nuoM	1747548	1749077	-	9	3	-0.48
FTA_2021	1841740	1842216	+	9	3	-0.48
dnaG	1005444	1007201	+	10	3	-0.52
tufA	1680897	1682021	-	10	3	-0.52
fusA	231523	233577	+	11	3	-0.56
FTA_2098	1121457	1121472	+	11	3	-0.56
FTA_2094	1292787	1292814	+	12	3	-0.60
FTA_1690	1528529	1529380	-	12	3	-0.60
FTA_1693	1531194	1531448	+	12	3	-0.60
FTA_1802	1629462	1630856	-	12	3	-0.60
FTA_1042	960762	961337	+	13	3	-0.64
leuS	1160412	1162793	-	13	3	-0.64
FTA_1300	1175943	1177037	-	13	3	-0.64
gshB	1224176	1225090	-	13	3	-0.64
nuoG	1753616	1755922	-	14	3	-0.67
lysS	1828933	1830603	-	14	3	-0.67
FTA_0627	581836	582408	+	15	3	-0.70
yajC	830445	830741	+	15	3	-0.70
kdsB	1328885	1329577	-	15	3	-0.70
ribH	72047	72430	-	16	3	-0.73
rpoA1	245968	246879	+	16	3	-0.73
FTA_1093	993480	993572	+	17	3	-0.75

gatA	1772108	1773493	-	17	3	-0.75
cmk	978413	979057	-	19	3	-0.80
pgi	1396618	1398180	-	19	3	-0.80
FTA_1578	1417380	1418579	+	19	3	-0.80
FTA_1664	1501728	1502387	-	19	3	-0.80
ftsY	1582660	1583631	-	20	3	-0.82
FTA_1091	992430	992507	+	21	3	-0.85
groEL	1641199	1642818	-	23	3	-0.88
FTA_0077	66583	66879	+	25	3	-0.92
rimM	1663718	1664167	-	25	3	-0.92
FTA_1689	1528240	1528458	-	31	3	-1.01
FTA_0748	699837	700865	-	42	3	-1.15
rpsF	984808	985110	+	0	2	0.30
FTA_1421	1277053	1277136	-	0	2	0.30
gmk	1320621	1321133	-	1	2	0.30
nuoB	1759701	1760117	-	1	2	0.30
FTA_0197	183291	183767	+	3	2	-0.18
groES	1642871	1643098	-	3	2	-0.18
nuoC	1759054	1759644	-	3	2	-0.18
FTA_0428	374680	375207	+	4	2	-0.30
thyA	707330	708094	-	4	2	-0.30
FTA_1254	1136368	1137492	-	4	2	-0.30
accB	1516315	1516728	-	4	2	-0.30
fabG	1081787	1082470	-	5	2	-0.40
FTA_1565	1402112	1403134	+	5	2	-0.40
nuoF	1756000	1757214	-	6	2	-0.48
map	434974	435684	+	7	2	-0.54
FTA_2108	528830	528846	-	7	2	-0.54
FTA_1566	1403194	1404195	+	7	2	-0.54
FTA_2109	1638696	1638712	+	7	2	-0.54
FTA_1898	1720021	1720539	-	7	2	-0.54
rpmH	178004	178078	+	8	2	-0.60
FTA_0204	188261	189955	-	8	2	-0.60
folB	1207195	1207488	+	8	2	-0.60
FTA_0171	161503	162099	-	9	2	-0.65
rubA	841249	841359	-	9	2	-0.65
FTA_1088	990209	990574	+	9	2	-0.65
infA	1183719	1183877	-	9	2	-0.65
FTA_0706	661040	661750	-	10	2	-0.70
atpA	1726882	1728363	-	10	2	-0.70
frr	227260	227757	+	11	2	-0.74
tolB	316629	317888	+	12	2	-0.78
hemF	982375	983241	-	12	2	-0.78
murI	739817	740554	+	13	2	-0.81
ispG	854221	855366	+	13	2	-0.81

dxs	1026876	1028663	+	13	2	-0.81
fmt	1225154	1226035	-	13	2	-0.81
FTA_0099	84072	84653	+	14	2	-0.85
FTA_1692	1530476	1531048	-	14	2	-0.85
FTA_1521	1363656	1364579	+	15	2	-0.88
FTA_1633	1474639	1474701	-	16	2	-0.90
FTA_1634	1474771	1475511	+	16	2	-0.90
FTA_2112	416131	416146	+	17	2	-0.93
hemC	146835	147677	+	20	2	-1.00
accA	281229	282116	+	20	2	-1.00
pssA	313366	314127	+	21	2	-1.02
rpoD	1007286	1008959	+	21	2	-1.02
dacB	1003060	1004403	-	23	2	-1.06
gpmI	1413448	1414938	+	24	2	-1.08
FTA_2105	645754	645768	+	0	1	0.00
FTA_2117	1347473	1347489	-	0	1	0.00
rplJ	1677797	1678255	-	0	1	0.00
atpF	1728986	1729396	-	0	1	0.00
rplP	237839	238192	+	1	1	0.00
rpsQ	238465	238656	+	1	1	0.00
rplF	240824	241300	+	1	1	0.00
tolR	315393	315671	+	1	1	0.00
rplM	1135801	1136169	-	1	1	0.00
FTA_2100	1347388	1347404	-	1	1	0.00
rpsO	1463339	1463545	-	1	1	0.00
atpB	1729858	1730589	-	1	1	0.00
ribD	74302	75309	-	2	1	-0.30
rpsG	231035	231448	+	2	1	-0.30
rpsS	236524	236742	+	2	1	-0.30
rpsC	237168	237779	+	2	1	-0.30
rpsD	245290	245850	+	2	1	-0.30
FTA_1124	1019560	1020231	+	2	1	-0.30
rplU	1378843	1379097	-	2	1	-0.30
FTA_2110	1742114	1742130	-	2	1	-0.30
rpsB	224867	225526	+	3	1	-0.48
tsf	225608	226417	+	3	1	-0.48
rplN	238808	239116	+	3	1	-0.48
FTA_1238	1125783	1126646	-	3	1	-0.48
FTA_2107	1347567	1347582	-	3	1	-0.48
ubiE	373926	374618	+	4	1	-0.60
bioB	1212803	1213684	+	4	1	-0.60
atpH	1728442	1728906	-	4	1	-0.60
nuoE	1757281	1757709	-	4	1	-0.60
pyrH	226481	227170	+	5	1	-0.70
glyA	694183	695376	-	5	1	-0.70

sufC	1177101	1177790	-	5	1	-0.70
infC	1332997	1333458	-	5	1	-0.70
FTA_2083	1410115	1410130	-	5	1	-0.70
FTA_2103	1888740	1888756	-	5	1	-0.70
FTA_0194	181336	182199	+	6	1	-0.78
rplO	242463	242834	+	6	1	-0.78
rpsR	985186	985344	+	6	1	-0.78
mraY	1539302	1540339	-	6	1	-0.78
uppS	227901	228563	+	7	1	-0.85
purK	369438	370475	+	7	1	-0.85
FTA_0580	531539	532774	-	7	1	-0.85
sufB	1177889	1179274	-	7	1	-0.85
FTA_1755	1583688	1584944	-	7	1	-0.85
hemA	1648586	1649776	-	7	1	-0.85
psd	427046	427837	-	8	1	-0.90
dxr	518396	519493	+	8	1	-0.90
FTA_0585	536206	536832	+	8	1	-0.90
rpsP	1664270	1664458	-	8	1	-0.90
rplA	1678485	1679123	-	8	1	-0.90
FTA_2093	416012	416043	+	9	1	-0.95
lgt	708139	708885	-	9	1	-0.95
murD	1538052	1539242	-	10	1	-1.00
ubiG	1565307	1565942	+	10	1	-1.00
serS	1415013	1416233	+	12	1	-1.08
ppa	1016872	1017333	+	13	1	-1.11
FTA_0690	639146	639835	+	15	1	-1.18
FTA_1671	1510390	1511112	+	15	1	-1.18
FTA_0477	426738	427013	+	16	1	-1.20
dnaX	1832043	1833620	+	17	1	-1.23
lpxC	1833694	1834494	-	17	1	-1.23
mnmA	69353	70372	+	22	1	-1.34
rplI	985414	985818	+	43	1	-1.63
rplD	234744	235307	+	0	0	0.00
rplX	239198	239455	+	0	0	0.00
rpmD	242271	242396	+	0	0	0.00
fabZ	523623	524054	+	0	0	0.00
FTA_2102	645844	645857	+	0	0	0.00
acpP	1081406	1081684	-	0	0	0.00
FTA_2090	1347285	1347300	-	0	0	0.00
FTA_2106	1682230	1682243	-	0	0	0.00
FTA_2111	1682305	1682329	-	0	0	0.00
FTA_2116	1888821	1888836	-	0	0	0.00
rpsJ	233658	233915	+	1	0	0.00
rplV	236818	237093	+	1	0	0.00
FTA_2091	638427	638442	-	1	0	0.00

rplL	1677358	1677675	-	1	0	0.00
FTA_2115	1682126	1682141	-	1	0	0.00
atpE	1729502	1729747	-	1	0	0.00

FTA_2089	158254	158268	+	2	0	-0.30
FTA_2200	238252	238392	+	2	0	-0.30
rplE	239527	240006	+	2	0	-0.30
rpsN	240085	240330	+	2	0	-0.30
rpsE	241764	242204	+	2	0	-0.30
rpsM	244484	244780	+	2	0	-0.30
rpsK	244879	245208	+	2	0	-0.30
FTA_1122	1018040	1018606	+	2	0	-0.30
rpmF	1085725	1085847	-	2	0	-0.30
FTA_1620	1460845	1461135	-	2	0	-0.30
rplK	1679183	1679569	-	2	0	-0.30
secE	1680235	1680645	-	2	0	-0.30
FTA_1893	1716128	1716436	-	2	0	-0.30
FTA_1928	1751182	1751454	-	2	0	-0.30
nuoA	1760168	1760506	-	2	0	-0.30
rpsL	230622	230936	+	3	0	-0.48
rplC	234079	234651	+	3	0	-0.48
rplW	235364	235603	+	3	0	-0.48
tolA	315718	316569	+	3	0	-0.48
FTA_2085	645923	645951	+	3	0	-0.48
rpmI	1332757	1332894	-	3	0	-0.48
rpsH	240407	240745	+	4	0	-0.60
secY	242905	244170	+	4	0	-0.60
FTA_2084	608918	608944	+	4	0	-0.60
tpiA	1706592	1707293	-	4	0	-0.60
lpxA	524117	524836	+	5	0	-0.70
plsX	1084580	1085566	-	5	0	-0.70
grpE	1138800	1139327	+	5	0	-0.70
lspA	400300	400704	-	6	0	-0.78
rplT	1332365	1332661	-	6	0	-0.78
FTA_2114	1458961	1458976	-	6	0	-0.78
FTA_2022	1843362	1843466	+	6	0	-0.78
rplQ	246984	247361	+	8	0	-0.90
rpmB	503415	503591	-	8	0	-0.90
FTA_1610	1452186	1452416	-	8	0	-0.90
FTA_2099	292953	292969	-	9	0	-0.95
FTA_0805	753935	754306	+	9	0	-0.95
dnaA	31	1446	+	10	0	-1.00
FTA_0865	803576	803638	+	10	0	-1.00
hemE	1742264	1743238	-	12	0	-1.08
gatC	1773562	1773783	-	13	0	-1.11
ndk	1247814	1248176	-	28	0	-1.45
FTA_1347	1214843	1215520	+	39	0	-1.59

* Gene annotations are from strain *Francisella tularensis ssp holarctica* FTNF002-00
