# Supplementary material for: A New Mechanism for Ribosome Rescue Can Recruit RF1 or RF2 to Nonstop Ribosomes
Source: mBio. 2018 Dec 18;9(6):e02436-18. doi: 10.1128/mBio.02436-18 (PMC6299226; doi:10.1128/mBio.02436-18)
Supplement: TABLE S2 [file mbo006184222st2.pdf]

Table S2: Bacterial strains with homologue to ArfT.

Strain	Genomic locus	Nucleotide identity to ArfT (%)	Amino acid identity to ArfT (%)
Francisella tularensis subsp. tularensis Schu S4	1554970-1555089	98	100
Francisella tularensis subsp. tularensis strain NR-21736	1152710-1152829	98	100
Francisella tularensis subsp. tularensis strain NR-21734	1152150-1152269	98	100
Francisella tularensis subsp. tularensis strain NR-21737	1151944-1152963	98	100
Francisella tularensis subsp. tularensis strain SCHU S4 NR-28534	1152614-1152733	98	100
Francisella tularensis strain Schu4 Mut-127	1254847-1254966	98	100
Francisella tularensis subsp. tularensis strain WY-00W4114	1228710-1228829	98	100
Francisella tularensis subsp. tularensis strain WY96-3418	1113660-1113779	98	100
Francisella tularensis subsp. tularensis strain T01	210237-210356	98	100
Francisella tularensis subsp. tularensis strain NIH B-38	1376736-1376855	98	100
Francisella tularensis subsp. holarctica LVS	1526352-1526471	100	100
Francisella tularensis subsp. holarctica FTNF002-00	803546-803665	100	100
Francisella tularensis subsp. holarctica strain OSU18	805432-805551	100	100
Francisella tularensis subsp. holarctica strain F92	799648-799767	100	100
Francisella tularensis subsp. holarctica strain 425	793290-793409	100	100
Francisella tularensis subsp. holarctica strain FSC200	803761-803880	100	100
Francisella tularensis subsp. holarctica strain OR96-0246	1199364-1199483	100	100
Francisella tularensis subsp. holarctica strain VT68	1028832-1028951	100	100
Francisella tularensis subsp. holarctica strain PHIT-FT049	798309-798428	100	100
Francisella tularensis subsp. novicida strain TCH2015	941649-941768	95	93
Francisella tularensis subsp. novicida strain Fx1	1186671-1186790	95	90
Francisella tularensis subsp. novicida strain D9876	1601327-1601446	95	90
Francisella tularensis subsp. novicida strain PA10-7858	1232777-1232896	60	88
Francisella tularensis subsp. novicida strain U112	943832-943951	60	88
Francisella tularensis subsp. novicida strain AZ06-7470	632257-632376	60	88
Francisella tularensis subsp. novicida strain DPG 3A-IS	836266-836370	60	89
Francisella tularensis subsp. novicida strain F6168	1446496-1446600	60	89
Francisella tularensis subsp. novicida strain AL97-2214	1192749-1192749	60	89
Francisella tularensis subsp. mediasiatica strain FSC147	1399724-1399828	98	100
Francisella hispaniensis strain FSC454	1317061-1317165	69	89
