# Supplementary figures and images for: A New Mechanism for Ribosome Rescue Can Recruit RF1 or RF2 to Nonstop Ribosomes
Source: mBio. 2018 Dec 18;9(6):e02436-18. doi: 10.1128/mBio.02436-18 (PMC6299226; doi:10.1128/mBio.02436-18)

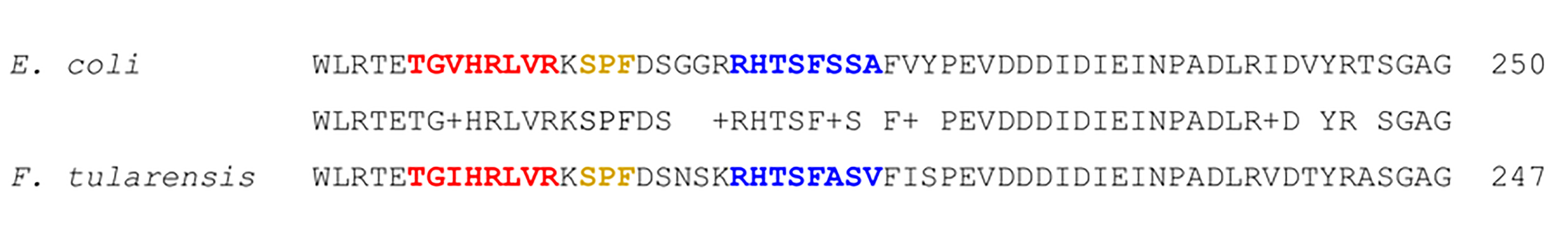

Supplement: FIG S3 [file mbo006184222sf3.tif]
