# Supplementary material for: A New Mechanism for Ribosome Rescue Can Recruit RF1 or RF2 to Nonstop Ribosomes
Source: mBio. 2018 Dec 18;9(6):e02436-18. doi: 10.1128/mBio.02436-18 (PMC6299226; doi:10.1128/mBio.02436-18)
Supplement: TABLE S3 [file mbo006184222st3.pdf]

Table S3: Strains, plasmids, and primers used in this study.

Strain, plasmid, or primer	Description	Reference or source
**Strains**		
wt LVS	*F. tularensis* ssp. holarctica live vaccine strain	Albany Medical College
ssrA::LtrB-A147	ssrA disabled by group II intron insertion	21
wt LVS pssrA	wt LVS complimented with ssrA on a plasmid	This study
wt LVS pArfT	wt LVS complimented with ArfT on a plasmid	This study
A147 pssrA	A147 complimented with ssrA on a plasmid	This study
A147 pArfT	A147 complimented with ArfT on a plasmid	This study
TG001	*E. coli* BL21(DE3) carrying pET28ArfT	This study
TG002	*E. coli* BL21(DE3) carrying pET28RF1	This study
TG003	*E. coli* BL21(DE3) carrying pET28ARF2	This study
TG004	in frame deletion of ArfT in wt LVS strain	This study
TG005	in frame deletion of FTA_0993 in wt LVS strain	This study
TG006	in frame deletion of ArfT in A147 pssrA strain	This study
TG007	in frame deletion of FTA-0993 in A147 pssrA strain	This study
TG008	in frame deletion of FTA-0993 in A147 strain	This study
**Plasmids**		
Himar H3	contains transposon for insertion into *F. tularensis* genome	29, 30
pKK214-MCS4	used for expression/complimentation in *F. tularensis*	32
pFtssrA	overexpression/complimentation of ssrA from Bfr promoter	This study
pArfT	overexpression/complimentation of ArfT from Bfr promoter	This study
pET28ArfT	overexpression of ArfT from T7 promoter	This study
pET28RF1	overexpression of *F. tularensis* RF1 from T7 promoter	This study

pET28RF2	overexpression of *F. tularensis* RF2 from T7 promoter	This study
pMP812	SacB allelic exchange vector	31
pMP812-ΔArfT	SacB allelic exchange vector to achieve in frame deletion of ArfT	This study
pMP812-Δ0993	SacB allelic exchange vector to achieve in frame deletion of ArfT	This study
**Primers**	**Sequence (5'-3')**	**Description**
ArfT_UF	GCGGTCGACGCGTTCAGTAGAGTAA CTTTCAGGAAAT	Upstream flank in ArfT deletion construct
ArfT_UR	GCGGGATCCGCGAATCAATTCTCCC CTTACTTTTGTT	Upstream flank in ArfT deletion construct
ArfT_DF	GCGGGATCCGCGTAATAAAATAAATT TTTGACAATTT	Downstream flank in ArfT deletion construct
ArfT_DR	ATTGCGGCCGCATTGGGATAATGAT CCTCTTCTCAA	Downstream flank in ArfT deletion construct
0993_UF	TATTAAGTCGACCTTTCAGGTAAGAC ATCAGCAG	Upstream flank in FTA_0993 deletion construct
0993_UR	TATTAAGGATCCAACGTTGATATTAT GTTGAAAAGCATTTCATT	Upstream flank in FTA_0993 deletion construct
0993_DF	TATTAAGGATCCTAGGATGTATGAAA CTATTGAGCATGG	Upstream flank in FTA_0993 deletion construct
0993_DR	ATTATGCGGCCGCTGGCATTATTGC TATTGATATATTCACTG	Upstream flank in FTA_0993 deletion construct
ArfT_CF	GCGGGATCCGCGATGGCTTACAATG AAAAAAATAGTTCA	ArfT plasmid complementation
ArfT_CR	GCGGAATTCGGATTATTTTTTGTTTG TAGAACCACCCTT	ArfT plasmid complementation
FtssrA_CF	GGATCCCGGCAATTTATGTTTTGTTG	ssrA plasmid complementation
FtssrA_CR	GCGGAATTCATAACTAGAGGAGAAG AGCTAGA	ssrA plasmid complementation
Bfr_F	GCTCGTCTAGAGATCCATACCCATG ATGGTTACTATTG	Bacterioferritin Promoter for plasmid complimentation
Bfr_R	GCCGCGGGATCCTATTGTTACCTCC ATTATTTAAAACTCTAATCA	Bacterioferritin Promoter for plasmid complimentation
ArfT_PF	GCGCATATGGGAATGGCTTACAATG AAAAAAATAGTTCA	ArfT protein expression construct
ArfT_PR	TTATTTTTTGTTTGTAGAACCACCCTT GGACTCGAGGCG	ArfT protein expression construct
RF1_PF	GCGCATATGGGAATGAAAGATTCTAT TAAAGCAAAATTGC	RF1 protein expression construct
RF1_PR	GCGTTACTCATCAGACATGGTTGCTA AAAGATC	RF1 protein expression construct
RF2_PF	GCGCATATGGGAATGGAGCTAGAAG ATGGTTCTATTTGGGAT	RF2 protein expression construct
RF2_PR	GCGTTATAACCCACTTTTTAAGCTAG CCTCAAT	RF2 protein expression construct

ArfT_KOF	CATCGTAACAGTTACAAACAACCC	For screening ArfT deletion
ArfT_KOR	GCTGGTATCACACTAGGTCAGAAC	For screening ArfT deletion
0993_KOF	AGCAAATACCAAACAACGATAACTGCT	For screening FTA_0993 deletion
0993_KOR	TCTTTTAATTAATTGCTCAAGCTTTTGGGCA	For screening FTA_0993 deletion
ssrA_QF	TCTAAGGTGCATGCCGAGGA	For qPCR amplification of tmRNA template
ssrA_QR	AACCCGCGTCCACAAACTCT	For qPCR amplification of tmRNA template
